# A Genotype–Phenotype Analysis of Glutathione Peroxidase 4 in Human Atrial Myocardium and Its Association with Postoperative Atrial Fibrillation

**DOI:** 10.3390/antiox11040721

**Published:** 2022-04-06

**Authors:** Islam A. Berdaweel, Alexander A. Hart, Andrew J. Jatis, Nathan Karlan, Shahab A. Akhter, Marie E. Gaine, Ryan M. Smith, Ethan J. Anderson

**Affiliations:** 1 Department of Pharmaceutical Sciences and Experimental Therapeutics, College of Pharmacy, University of Iowa, Iowa City, IA 52242, USA; islam-berdaweel@uiowa.edu (I.A.B.); jatis.andrew@mayo.edu (A.J.J.); nkarlan@bozemanhealth.org (N.K.); marie-gaine@uiowa.edu (M.E.G.); smith_ryan_matthew@lilly.com (R.M.S.); 2 Roy J. and Lucille A. Carver College of Medicine, University of Iowa, Iowa City, IA 52242, USA; alexander-hart@uiowa.edu; 3 Department of Cardiovascular Sciences, Brody School of Medicine, East Carolina Heart Institute, Greenville, NC 28592, USA; akhtersh16@ecu.edu; 4 Fraternal Order of Eagles Diabetes Research Center, University of Iowa, Iowa City, IA 52242, USA

**Keywords:** postoperative atrial fibrillation, glutathione peroxidase-4, biomarkers, reactive oxygen species, single nucleotide variants

## Abstract

Heterogeneity in the incidence of postoperative atrial fibrillation (POAF) following heart surgery implies that underlying genetic and/or physiological factors impart a higher risk of this complication to certain patients. Glutathione peroxidase-4 (GPx4) is a vital selenoenzyme responsible for neutralizing lipid peroxides, mediators of oxidative stress known to contribute to postoperative arrhythmogenesis. Here, we sought to determine whether *GPX4* single nucleotide variants are associated with POAF, and whether any of these variants are linked with altered *GPX4* enzyme content or activity in myocardial tissue. Sequencing analysis was performed across the *GPX4* coding region within chromosome 19 from a cohort of patients (N = 189) undergoing elective coronary artery bypass graft (−/+ valve) surgery. GPx4 enzyme content and activity were also analyzed in matching samples of atrial myocardium from these patients. Incidence of POAF was 25% in this cohort. Five *GPX4* variants were associated with POAF risk (permutated *p* ≤ 0.05), and eight variants associated with altered myocardial GPx4 content and activity (*p* < 0.05). One of these variants (rs713041) is a well-known modifier of cardiovascular disease risk. Collectively, these findings suggest *GPX4* variants are potential risk modifiers and/or predictors of POAF. Moreover, they illustrate a genotype–phenotype link with this selenoenzyme, which will inform future mechanistic studies.

## 1. Introduction

Atrial fibrillation (AF) is a dangerous cardiac condition that is rapidly rising in incidence and prevalence worldwide, approaching epidemic proportions in recent decades [1]. Postoperative AF (POAF) is a clinical complication consistently reported after cardiac surgery (usually reported during the first 2–3 days), and develops in about 20–40% of patients after coronary artery bypass graft surgery (CABG) and up to 60% of patients after valve repair/replacement surgeries [2]. Importantly, POAF cannot be viewed simply as a short-term complication; in fact it is linked with a considerable increase in morbidity and mortality. Patients who develop POAF have higher incidence of congestive heart failure, stroke, renal insufficiency, and up to 10-fold higher risk for future AF. They also have prolonged hospitalization and higher rehospitalization rates, which translates into an extra pressure on the healthcare system [1,2,3,4,5].

Although POAF is a well-recognized clinical problem, the underlying mechanisms are still poorly understood. Among the proposed mechanisms, atrial oxidative stress and systemic inflammation are postulated as major arrhythmogenic factors in POAF, a conclusion that is supported by the results of several studies using atrial samples obtained during the perioperative window [2,6,7]. Specifically, lipid peroxidation and the downstream reactive carbonyl species (RCS) formed from their breakdown (e.g., 4-hydroxynonenal(4-HNE) seem to play a significant pathogenic role in POAF [8].

In a recent study, patients who developed POAF were found to have pre-existing abnormalities in mitochondrial function, and underlying inflammation and oxidative stress in their myocardium, as compared with patients who maintained sinus rhythm [9]. This mitochondrial dysfunction was found to be associated with higher atrial tissue levels of 4-HNE in patients who developed POAF [10]. Another report demonstrated that activity of NADPH oxidase, a superoxide free radical producing enzyme, was significantly higher in patients who developed POAF; authors further concluded NADPH oxidase activity is an independent predictor for POAF [11]. Likewise, our group has shown that POAF risk is strongly associated with monoamine oxidase (MAO) activity in atrial myocardium [12]. MAO is a major source of oxidative stress in cardiomyocytes and contributes to the pathogenesis of numerous cardiac diseases [13]. Additionally, we found that incidence of POAF was inversely associated with glutathione peroxidase (GPx) and total glutathione (GSHt) content in the atrial tissue, suggesting that antioxidant capacity does play a role in mitigating POAF. 

Intracellular levels of reactive oxygen species (ROS) are tightly controlled through a vast network of antioxidant enzymes that have certain subcellular compartmental and substrate specificity. Among these antioxidant enzymes is the selenoenzyme GPx4, a 20–34 kDa monomer and a member of the glutathione peroxidase superfamily. GPx4 is considered a unique antioxidant enzyme due to its ability to inhibit membrane lipid peroxidation and ultimately reduce the levels of RCS [14]. A number of factors are known to regulate GPx4 at the transcriptional, translational, and post-translational level. Intracellular GHS depletion induced by ROS accumulation or by cystine depletion (e.g., inhibiting system Xc-cysteine/glutamate transporter) has been shown to inhibit GPx4 activity and enhance ferroptosis [15,16]. Selenium availability is another factor that is essential for optimum GPx4 activity [17,18,19]. At the translational level, the incorporation of selenocysteine (Sec) in the enzyme active site is necessary for proper GPx4 assembly and maturation. This maturation step is regulated by an isopentenylated Sec-tRNA^[sec]ser^, a biochemical product which requires an isopentenyl transferase enzyme, an isopentenyl pyrophosphate (IPP), and a mevalonate pathway byproduct as substrate [20]. Thus, modulation of these two components can impact GPx4 expression and activity. At the post-translational level, ubiquitination and acetylation are modifications (PTMs) which have been shown to regulate GPx4 activity and/or stability [21,22,23,24]. 

GPx4 has been intensively studied in recent years due to its important role as a regulator of ferroptosis, the form of cell death caused by excessive lipid peroxidation [25,26,27]. Previous reports have shown that *GPX4* variants are associated with cardiovascular diseases, particularly rs713041 [28,29,30,31]. However, despite the known link between oxidative stress and POAF, no studies have addressed whether there is a link between GPx4 and POAF. Moreover, given the high complexity of transcriptional and post-transcriptional regulation of this selenoenzyme [19,32], it is important to ascertain if *GPX4* variants which have previously been associated with cardiovascular disease (e.g., rs713041) influence GPx4 enzyme content or activity.

In this study, we tested the hypothesis that *GPX4* variants are associated with POAF, and we also sought to determine if any variants were linked to altered GPx4 enzyme level and/or activity in human myocardium. To accomplish these objectives, we performed a genotype–phenotype analysis of GPx4 in samples of myocardium dissected from right atrial appendages collected from a cohort of patients undergoing non-emergent CABG and assessed the relationship between these variables and incidence of POAF. 

## 2. Materials and Methods

### 2.1. Study Design and Tissues Used for Analysis

**Figure 1:**
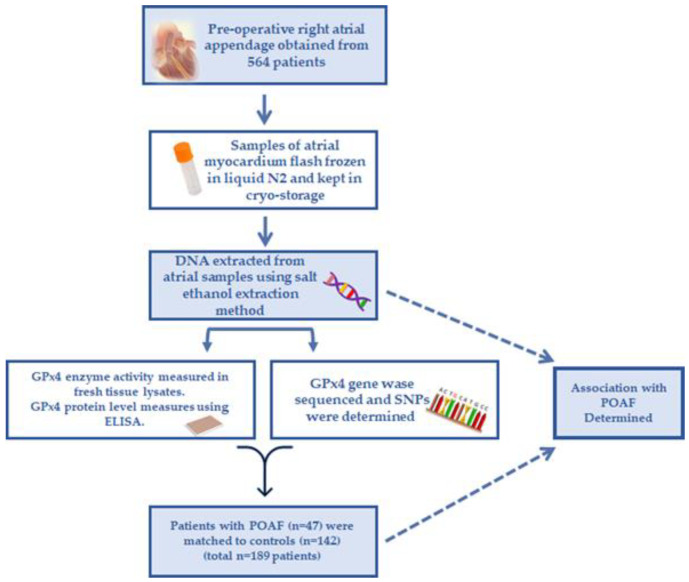
Overall study flow and design. Right atrial appendage samples were collected intra-operatively from patients undergoing non-emergent CABG. Salt ethanol method was used extract DNA from these samples. The genomic region encoding *GPX4* was sequenced using Ion AmpliSeq (ThermoFisher) according to methods described in Methods section. GPx4 enzyme content and activity were measured as described in the Methods section. CABG: coronary artery bypass graft.

All aspects of this study received approval from Institutional Review Boards of East Carolina University and the University of Iowa. Overall study design and workflow is shown in Figure 1. Patients undergoing primary on-pump, non-emergent coronary artery bypass graft (CABG) or CABG + valve surgery between January 2014 and December 2018 were screened prior to surgery, and informed consent was obtained from 594 patients. Routine concurrent procedures such as pericardiectomy, repair or restoration of the heart or pericardium, transmyocardial laser revascularization, coronary endarterectomy, internal cardiac defibrillation, femoral-femoral cardiopulmonary bypass, pacemaker insertion, or pericardiectomy were included in this cohort. Patients > 75 years old and/or with history of cardiac surgery were excluded from this study, as were patients undergoing surgical or catheter maze procedures and those with preoperative shock. A fasted blood sample was obtained from each patient preoperatively on the morning of surgery, before onset of anesthesia. Prior to institution of cardiopulmonary bypass, a sample of the right atrial appendage (RAA) was resected in the region of the purse-string suture and endocardium was immediately rinsed in ice-cold Buffer X [33,34], trimmed of epicardium and fat, and a portion rapidly frozen in liquid N2. This method ensured that all atrial tissue samples were uniformly and rapidly processed to minimize protein and RNA degradation.

### 2.2. Analyzing Genotype–Phenotype and POAF Risk

Patients’ heart rate and rhythm were continuously monitored throughout the perioperative window with telemetry until discharge. POAF was defined by a sustained episode of atrial fibrillation lasting ≥1 min postoperatively, or for any length of time requiring intervention for hemodynamic compromise. In this nested case control study, 47 patients who had POAF without prior history of AF (30 of 47) or a history of AF (17 of 47) were selected as cases. Our rationale for including patients with history of AF in our analysis was that (1) many patients undergoing elective CABG and/or CABG+ valve surgery have a history of AF, and (2) patients with history of AF are at far greater risk for POAF [6,35]. Propensity score matching was then used to identify 142 patients that did not have a history of atrial fibrillation or any POAF as our control sinus rhythm (SR) group. This SR group was matched to the greatest extent possible for age, body mass index (BMI), sex, race, cardiovascular disease, and other comorbidities known to be linked to POAF.

### 2.3. DNA Extraction and Storage

Salt–ethanol precipitation method was used to extract DNA from cryo-stored atrial tissue samples. In brief, samples were initially digested with proteinase K in nuclei lysis buffer in a 55 °C water bath overnight. The following day, proteinase K was re-added, vortexed briefly, and samples then put back into the water bath for 1 h. NaCl was then added, thoroughly mixed, and place into a 4 °C refrigerator for an additional hour. The sample was then centrifuged at 13,000 rpm for 5 min. Supernatant was then added to 1 mL 95% ethanol in a sterile centrifuge tube and centrifuged at 2000 rpm. The precipitated DNA remaining at the bottom of the tube was then washed with 70% ethanol and centrifuged again. TE buffer (100 μL) was then added to the precipitated DNA and stored at −80 °C.

### 2.4. Myocardial Protein Preparation

Small slices of frozen myocardial tissues (20–30 mg) were used. Tissue slices were placed in 200 µL ice-cold TEE buffer (containing 10 mM Tris, 1 mM EDTA, 1 mM EGTA, pH 7.4), 0.3 mM M_2_VP, and 1% (*v*/*v*) Tween-20. Samples were homogenized using a glass grinder (Kimble Chase, Vineland, NJ, USA) and lysates were centrifuged at 10,000× *g* for 15 min at 4 °C. A portion of the supernatant was then used immediately for GPx4 activity assay, while the remaining amounts were mixed with a protease inhibitor cocktail (Roche) and frozen at −20 °C for GPx4 protein quantification. 

### 2.5. Myocardial GPx4 Activity Analysis

GPx4 activity assays were performed immediately on fresh myocardial tissue lysate using phosphatidylcholine hydroperoxide (PCOOH), a common phospholipid peroxide and GPx4 substrate. Not all samples which were genotyped were also processed for GPx4 activity as the assay required ≥10 mg of tissue, and many samples were depleted because of DNA extraction and use in prior studies [36,37]. PCOOH was synthesized based on a previously published protocol [38].

Briefly, a buffer composed of 0.2 M Tris-base and 0.3 mM sodium deoxycholate (pH 7.4) was used for the reaction. A solution of 5 mg L-α-phosphatidylcholine Type III/S (Sigma-Aldrich, St. Louis, MO, USA, CAS# 8002-43-5) in chloroform was slowly added to 2 mL of the reaction buffer while stirring using a magnetic stirrer. This generated a milky emulsion to which was added another 18 mL of the reaction buffer. Peroxidation of the phospholipids in solution was then started by adding 250,000 U of lipoxidase type V from soybean (Sigma-Aldrich, CAS# 9029-60-1). Solution was then mixed for 30 min at room temperature with continuous stirring and bubbled oxygen. The PCOOH solution was then passed through a Sep-Pak C18 cartridge (Waters, Milford, MA, USA, Part No. WAT023635), previously primed with 4 mL of methanol and equilibrated by 40 mL of DDI water. After subsequent washes with reaction buffer, PCOOH was eluted from the column using 2 mL of methanol. The concentration of PCOOH in the methanol was then determined by measuring absorbance at 234 nm using a UV spectrophotometer (Shimadzu, Kyoto, Japan) and calculated using the following equation: [PCOOH] (mM) = (Abs_234_/25) × 50 (using 1 cm cuvette) × dilution factor) [30]. The resulting PCOOH solution was stored in a dark glass bottle at −80 °C and was stable for up to 1 week for repeated use. 

Activity assays were carried out in a 96-well plate using an assay buffer composed of 100 mM Tris-base, 2 mM EDTA, 1.5 mM NaN_3_, 0.1% (*v*/*v*), peroxide free Triton X-100, pH 7.4, and stock solutions of 15 U/mL glutathione disulfide reductase (GR) (Sigma-Aldrich: G3664), 30 mM glutathione (GSH) (Sigma-Aldrich), and 2 mM nicotinamide adenine dinucleotide phosphate (NADPH). All these solutions were freshly prepared and kept on ice during the assay. A reaction mixture composed of 60 µL of the assay buffer, 10 µL GR, 10 µL GSH, 5 µL NADPH, and 15 µL of tissue lysate were added to each well, then the plate was incubated for 8 min at 37 °C with shaking to allow redox equilibration to occur in the samples. GPx4 activity was then initiated by adding 30 µM PCOOH. GPx4 activity was determined to be the PCOOH-dependent loss of NADPH fluorescence (360 ex/450 em) over 30 min in a multi-well plate-reader fluorometer (Agilent BioTek, Inc. Santa Clara, CA, USA.). A paired sample of identical lysate was run in parallel with all components except PCOOH, to determine the GPx4-independent background rate (i.e., negative controls). 

### 2.6. Myocardial GPx4 Enzyme Quantitation

A custom enzyme-linked immunosorbent assay (ELISA) developed by our group was used to determine GPx4 protein concentration in the myocardial tissue samples. Standard curves of GPx4 were generated using recombinant human Gpx4 (Abcam, Boston, MA, USA, ab82660) diluted in 1 X- Dulbecco’s phosphate buffered saline (DPBS, Gibco, cat# 14190144). Samples of recombinant GPx4 and myocardial tissue lysate were incubated in each well of an immobilon-coated 96-well plate (ThermoFisher Scientific, Waltham, MA, USA) overnight at 4 °C. Blocking was then performed using 5% bovine serum albumin (BSA) for 2 h at 37 °C. Goat anti-GPx4 polyclonal primary antibody (Abcam, ab116703) was then diluted in DPBS + 0.1% BSA and the wells incubated overnight at 4 °C. The wells were then washed and incubated with horseradish peroxidase-conjugated rabbit anti-goat IgG (Bio-Rad, Inc., Hempstead, UK) at 37 °C for 2 h. Amplex Ultra-red solution (Thermo-Fisher Scientific, Waltham, MA, USA, A12222) was then added to each well, and fluorescence of resorufin was detected in each well using a plate-reader fluorometer (BioTek Synergy, Agilent BioTek, Inc., Santa Clara, CA, USA) at 530/595 nm excitation/emission after 15 min. 

### 2.7. DNA Sequencing 

A comprehensive panel of primers was used to amplify the genomic region encoding *GPX4* in a highly multiplexed reaction. This panel provided 95% coverage of *GPX4* exons and included the proximal promoter and upstream enhancer region, permitting the detection of common and rare single nucleotide variants (SNVs) proximal to *GPX4* region on chromosome 19. All variants with clinical implications previously identified in published reports were covered in the sequencing panel design. The primer panel was custom designed through the Ion AmpliSeq Designer tool (v7.02) and ordered through ThermoFisher (Waltham, MA, USA). See Supplemental Table S1 for amplicon design and primer coverage.

Sequencing libraries were constructed using the Ion AmpliSeq Library Kit 2.0, per manufacturer’s instructions. Individual samples were barcoded (Ion Xpress Barcode Adapters 1–96) and the Ion Library Equalizer Kit was used to multiplex 96 samples on each of two Ion 530 sequencing chips, loaded by an Ion Chef. The chips were subsequently sequenced on an Ion S5 sequencing system. Sequenced reads were aligned to hg19 using the Torrent Suite Software (v5.10.1), with a custom AmpliSeq hot spot panel for SNP identification. For sequencing metrics, see the run reports in the Supplemental Materials section.

### 2.8. Statistical Analysis

All preoperative clinical and demographic characteristics of these two groups were compared univariately using chi-squared test or two-sided *t*-tests for categorical and continuous variables, respectively. An alpha value of 0.05 was used for significance in all univariate analyses. All univariate analyses were completed using SAS version 9.4 (SAS Institute, Cary, NC, USA).

For multivariate analysis, logistic regression was run across all exons and 100 base pairs upstream and downstream of each exon for *GPX4* controlling for age, sex, race, BMI, smoking status, and past medical history of hypertension. The minimum genotypic frequency was set at 0.1, minor allele frequency 0.05, and Hardy Weinberg Equilibrium limit at 10 × $10^{-7}$. Recorded metrics include odds ratios (OR), unadjusted *p*-values, permutated *p*-values (with number of permutations), Benjamini–Hochberg procedure adjusted *p*-value, and False Discovery Rate Benjamini–Uekutieli procedure adjusted *p*-values. Permutated *p*-values, as well as other adjustments, were used to reduce the possibility of rejecting the null hypothesis by chance, rather than due to a statistically significant association between *GPX4* variants and POAF. Multivariate analysis was conducted using Plink version 1.9, adjusting for age, sex, race, body mass index (BMI), smoking status, and history of hypertension. Variants with either unadjusted or permutated *p*-values < 0.10 were recorded to present both statistically significant and marginally significant values.

## 3. Results

### 3.1. Patient Characteristics and Incidence of POAF

The clinical and demographic characteristics of the study population are summarized in Table 1 according to POAF status. A total of 189 patients were included in this study and 47 patients (24.8%) out of 189 patients developed POAF. Patients who had POAF were more likely to be older (*p* = 0.0447), have a history of AF (*p* < 0.0001) or heart failure (HF) (*p* = 0.0095), and have concordant left ventricular dysfunction (lower ejection fraction, EF) (*p* = 0.0073). All other baseline demographics, comorbidities, medications, and cardiac functions were similar between the two groups. Neither GPx4 protein levels nor maximal GPx4 activity were significantly associated with POAF in this cohort. 

### 3.2. Variants of GPX4 and POAF Risk 

To determine whether *GPX4* variants are associated with POAF risk, sequencing data were analyzed using PLINK, adjusting for age, sex, race, BMI, smoking status, and past medical history of hypertension. Two variants (rs2075710, rs8178977) were found to be associated with an increased risk for POAF, and three variants (rs2074452, rs3826961, rs3746162) were associated with decreased risk for POAF (Table 2). 

### 3.3. Myocardial GPx4 Content and Activity and Influence of GPX4 Variants

**Figure 2:**
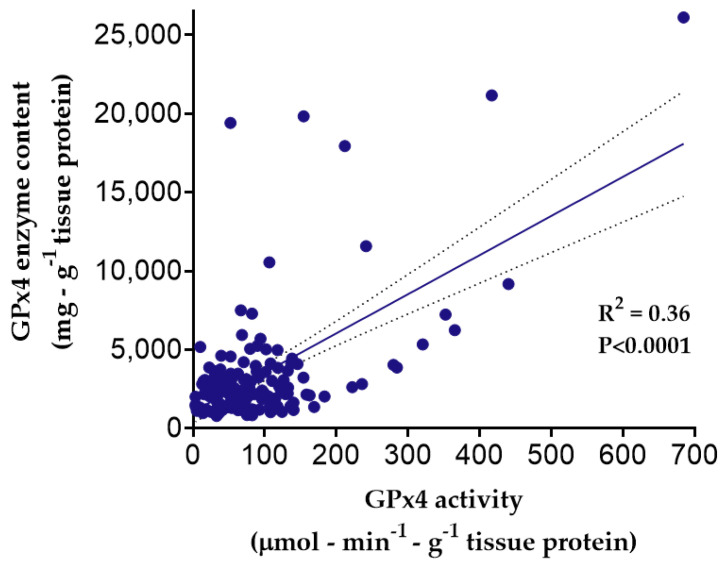
Relationship between GPx4 enzyme content and activity in human atrial myocardium. GPx4 enzyme activity (μmol·min^−1^·g^−1^ total protein) and total GPx4 enzyme (mg) were measured in fresh lysates prepared from samples of human atrial myocardium using protocols described in Methods section. Both enzyme activity and enzyme concentration values shown here are normalized against the total tissue protein (g). Each symbol corresponds to one individual patient (N = 189 patients). Statistical significance was calculated using simple linear regression, with dashed lines showing 95% confidence intervals.

Biochemical analysis of GPx4 in the myocardial tissue samples revealed that GPx4 enzyme content (i.e., expression) weakly correlated with maximal GPx4 activity in the tissue (R^2^ = 0.36, *p* < 0.0001), although a high degree of variability was observed (Figure 2). Interestingly, many samples had no detectable GPx4 activity despite the presence of the GPx4 enzyme (not shown), and these patients were not included in the phenotypic analysis. 

**Figure 3:**
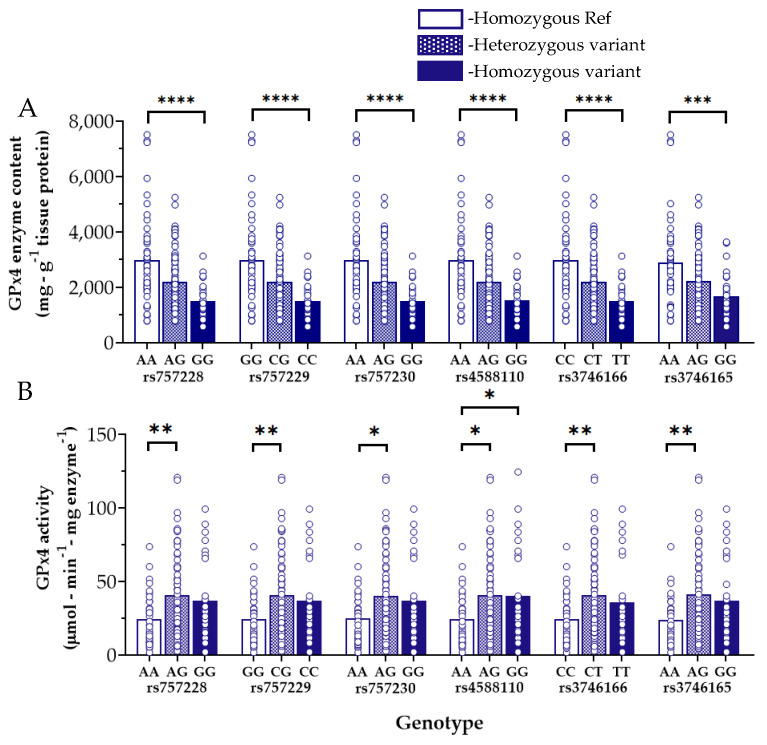
*GPX4* variants associated with decreased GPx4 enzyme content but increased activity in human atrial myocardium. Five distinct polymorphisms associated with decreased tissue levels of GPx4 enzyme (**A**) but increased GPx4 activity (**B**) are shown in the panels above. Each open circle represents one patient, open bars = Homozygous for the Referent nucleotide, hatched bars = Heterozygous for the variant nucleotide (i.e., one allele), filled bars = Homozygous for the variant nucleotide. **** *p* < 0.0001, *** *p* < 0.001, ** *p* < 0.01, * *p* < 0.05 for main effect between genotypes indicated.

We performed a GPx4 genotype–phenotype analysis within the myocardial tissue samples and found six *GPX4* variants which were significantly associated with substantially lower levels of myocardial GPx4 enzyme content as compared with the Referent nucleotide carriers (Figure 3A). Measuring both GPx4 content and activity in the same sample afforded us the ability to examine maximal GPx4 activity (in µmol/min) expressed per milligram of GPx4 enzyme, a more rigorous parameter for enzyme kinetic analysis then we showed in a previous report on MAO in human myocardium [37]. Intriguingly, despite the lower myocardial GPx4 enzyme levels in those patients carrying these particular *GPX4* variants, GPx4 activity was significantly higher in some of these carriers as compared with Referents (Figure 3B).

**Figure 4:**
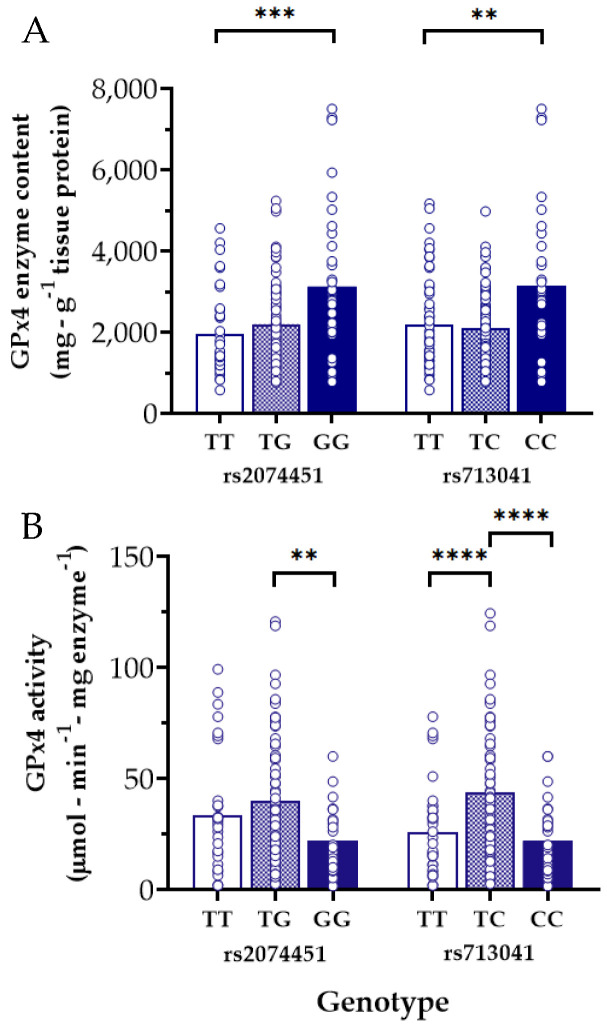
*GPX4* variants associated with increased GPx4 enzyme content but decreased activity in human atrial myocardium. Two distinct polymorphisms associated with increased tissue levels of GPx4 protein (**A**) but decreased GPx4 activity (**B**) are shown in the panels above. Each open circle represents one patient, open bars = Homozygous for the Referent nucleotide, hatched bars = Heterozygous for the variant nucleotide (i.e., one allele), filled bars = Homozygous for the variant nucleotide. **** *p* < 0.0001, *** *p* < 0.001, ** *p* < 0.01 for main effect between genotypes indicated.

We also observed the reciprocal situation, finding two *GPX4* variants which were associated with increased myocardial GPx4 enzyme content (Figure 4A) but had correspondingly lower GPx4 activity (Figure 4B) compared with Referent carriers. One of these variants, rs713041, has previously been linked in numerous reports to increased risk for cardiovascular diseases [29,30,31]. 

None of the variants which were associated with altered myocardial GPx4 content or activity were also associated with POAF in this cohort, although several have been associated with a small but significantly higher risk for AF in previous genome-wide association studies [39,40].

## 4. Discussion

Given the heterogeneity in the incidence and complex pathophysiology of POAF, research efforts directed at this postoperative complication should encompass a holistic approach which accounts for relevant variables, including patient genotype, corresponding phenotype, and also the patient’s clinical status (i.e., environment). While the exact pathogenesis of POAF is incompletely understood, the general consensus is that it likely involves an interplay between catecholaminergic, oxidative, and inflammatory stress in the patient during the postoperative period, acting in concert with the patient’s genotype [2,7,41,42,43]. The findings of this study align with this common agreement and for the first time point out a potential link between selenoenzyme GPx4 and POAF risk. Our results also show an association between several *GPX4* variants (rs2075710, rs2074452, rs3826961, and rs3746162) and POAF risk. We also report several *GPX4* variants which are associated with higher and lower levels of GPx4 content and/or activity, notably rs713041. It is important to point out that some of these variants have also been shown to be associated with changes in GPx4 mRNA expression across multiple tissues [44]. Although our findings show modest correlation between myocardial GPx4 content and activity (Figure 1), these findings also suggest that GPx4 enzyme activity is not always a direct reflection of enzyme expression (i.e., content) in myocardium.

Several previous studies have indicated that systemic oxidative stress and inflammation are major pathophysiological factors underlying POAF. Both factors have been shown to predispose patients to POAF via formation of an abnormal preoperative atrial substrate that makes patients more vulnerable to the acute intra- and postoperative stress and trauma [35,45,46,47,48]. Researchers have described several forms and degrees of atrial structural, mechanical, and electrical remodeling in patients with POAF. All of these forms of remodeling are impacted by factors such as age, history of diabetes, obesity, genetic variations, and increased oxidative stress and inflammation [41,43,49,50]. It is noteworthy that inflammation and oxidative stress are inextricably linked conditions [51,52]. Oxidative myocardial tissue damage and cell death occur either through direct oxidation of crucial cellular components such as DNA, lipids, and proteins, or as a result of lipid peroxidation. Moreover, this oxidative stress subsequently leads to over production of RCS, including lipid-derived aldehydes capable of irreversibly modifying critical cellular components [8,53,54]. Such self-propagating RCS could disturb various metabolic and signaling pathways and induce irreversible structural and functional changes in the myocardium [55]. 

The selenoenzyme GPx4 is distinct because it neutralizes membrane-bound and complex lipid hydroperoxides, thereby protecting against downstream RCS-related myocardial tissue damage. Biomarkers of lipid peroxidation (e.g., F2-isoprostanes, F3-isoprostanes, and isofurans) were found to be independently associated with the risk of POAF as reported by a multicenter prospective cohort study [56]. Likewise, a randomized controlled trial by Rodrigo and colleagues illustrated an inverse relationship between myocardial GPx activity, plasma RCS (e.g., malondialdehyde) levels, and the incidence of POAF [57]. This supports our findings that five *GPX4* variants are associated with POAF risk (Table 2). Among these variants, it is notable that rs2074451 was also found to be associated with chronic AF risk, according to two different genome wide association studies (GWAS) [39,40].

A cardioprotective role for GPx4 has been illustrated through several experimental models. GPx4 overexpression inhibited atherosclerotic lesions in the aortic sinus through mitigating vascular cell sensitivity to oxidized lipids [58]. Likewise, GPx4 overexpression was found to reverse cardiomyopathy in mouse models of diabetes by preventing mitochondrial damage [59]. Another report showed that GPx4 overexpression reduced myocardial ischemic injury by blunting formation of phospholipid hydroperoxides and subsequent damage to mitochondrial membranes [60]. 

Despite much experimental and clinical evidence supporting a protective role for GPx4 in the heart, very little is known concerning the impact of *GPX4* variants on the expression and/or activity of the enzyme. In this study, we were able to identify six variants associated with lower atrial GPx4 content, but paradoxically, higher GPx4 enzyme activity (Figure 3). Reciprocally, two other *GPX4* variants were found to be linked to higher atrial GPx4 levels with lower GPx4 enzyme activity (Figure 4). Among these, rs713041 is especially noteworthy due to its proven functional impact on GPx4 level and activity, as demonstrated by Méplan and colleagues, who found that this variant influences ribosome binding to the 3′UTR of the selenoprotein mRNAs, which is required for selenocysteine incorporation [61]. This change in selenocysteine affinity was found to significantly impact GPx4 protein levels as well as its enzymatic activity [61,62]. The decreased GPx4 enzyme activity seen with rs713041 carriers could explain, at least in part, the numerous reports showing the association of this variant with several cardiovascular diseases [28,29,30,31]. In the present study, we were unable to observe an association between rs713041 and POAF, which could be partially due to the small sample size of our study cohort. Alternatively, it is plausible that the effect of these variants only become evident when selenium is limiting, as it has been previously shown [63]. However, it should be emphasized that in larger cohorts, all of the variants we have identified in the present study have been found to be associated with a modest increase in AF risk in multiple GWAS [39,40,64,65], including the FinnGen GWAS study (https://www.finngen.fi/en, accessed on 13 January 2022). Additionally, an association between these variants and other cardiometabolic diseases, including diabetes and obesity, has also been reported in other genomic studies, further underscoring the important role of GPx4 in maintaining cardiometabolic homeostasis. 

Results of the present study also illustrate that GPx4 expression/content is not always directly proportional to its activity, as shown in Figure 3 and Figure 4. This strongly suggests that the enzyme is post-transcriptionally and post-translationally modified and regulated by as-yet unknown mechanisms. In addition to the role of selenium and selenocysteine incorporation as described previously (i.e., post-transcriptional modification), there are various post-translational modifications which could impact GPx4 stability and activity. Such modifications can alter the tertiary structure of the enzyme, its interaction with other proteins, and its subcellular localization [21]. It is also plausible that some of the variants we have identified might have a mixed effect on GPx4 expression and/or activity, which could result in higher expression and lower activity or vice versa, a possibility that requires further investigation. 

Collectively, our study is strengthened by several factors, specifically inclusion of a mixed cohort of patients (e.g., sex and race), and by combining genotypic and phenotypic analysis of GPx4. While the relatively low sample size is admittedly a limitation in this study, the high prevalence of POAF mitigates this shortcoming and allows us to discern an effect. Another limitation is that the lack of baseline selenium levels in these patients prevents us from knowing how this variable may have influenced the phenotypic differences seen here.

## 5. Conclusions

The findings of this study suggest that *GPX4* variants are potential risk modifiers for POAF, and that several variants are associated with altered GPx4 enzyme content and activity in the heart. Further investigation in experimental models will be necessary to elucidate the precise mechanisms by which these variants influence GPx4 expression and/or activity in the heart, and to further improve our understanding of GPx4 and lipid peroxidation in arrhythmogenesis.

## Figures and Tables

**Table 1:** Patient clinical and demographic characteristics.

Variables	POAF N (%)	Sinus Rhythm N (%)	*p*-Value
**Overall**	47 (25)	142 (75)	
**Demographics**			
**Age > 65 years**	33 (70.21)	76 (53.52)	0.0447
**White race**	42 (89.36)	113 (79.58)	0.1301
**Obese**	21 (44.68)	60 (42.25)	0.7707
**Male**	37 (78.72)	111 (78.17)	0.9363
**Smoking Status**	12 (25.53)	40 (28.17)	0.7257
**Comorbidities**			
**History of AF**	17 (36.17)	9 (6.34)	<0.0001
**COPD**	5 (10.64)	14 (9.86)	0.8776
**DM**	28 (59.57)	63 (44.37)	0.0705
**HF**	15 (31.91)	21 (14.79)	0.0095
**HTN**	41 (87.23)	119 (83.80)	0.5716
**Prior MI**	19 (40.43)	52 (36.62)	0.6405
**Medications**			
**ACEi/ARB**	21 (44.68)	56 (39.44)	0.5259
**β- blockers**	40 (85.11)	109 (76.76)	0.2247
**CCBs**	10 (21.28)	32 (22.54)	0.8572
**Diuretics**	24 (51.06)	59 (41.55)	0.2546
**DM Meds**	25 (53.19)	59 (41.55)	0.1638
**Nitrates**	28 (59.57)	92 (64.79)	0.5198
**Statins**	36 (76.60)	118 (83.10)	0.3198
**Cardiac Function**			
**EF > 35**	35 (74.47)	127 (90.07)	0.0073
**HR ≥ 70 bpm**	19 (40.43)	69 (48.59)	0.3306
**LAD ≥ 5 cm ^a^**	2 (4.26)	2 (1.41)	0.2442
**GPx4 values**	** Mean ± SD **		
***GPX4* activity ^a^**	129.7 ± 154.4	113.5 ± 134.7	0.5405
***GPX4* protein level ^a^**	2293.2 ± 1119.4	2507.5 ± 1542.3	0.3165

Sample size N = 189 patients; (POAF N = 47, sinus rhythm N = 142). ^a^ Missing N = 56, 41, 8 for LAD, GPx4 activity and GPx4 protein level, respectively. AF, atrial fibrillation; COPD, chronic obstructive pulmonary disease; DM, diabetes mellitus; HF, heart failure; HTN, hypertension; MI, myocardial infarction; ACEi, angiotensin converting enzyme inhibitors; ARB, angiotensin receptor blocker; CCB, calcium channel blocker; EF, ejection fraction; HR, heart rate; LAD, left atrial diameter.

**Table 2:** *GPX4* variants associated with POAF risk.

SNP	Minor Allele	Consequence	OR ^a^	*p*-Value ^b^	Permutated *p*-Value	Number of Permutations	FDR_BH ^c^	FDR_BY ^d^
** rs2075710 **	T	Regulatory region	1.869	0.0206	0.0199	1006	0.0965	0.3137
** rs8178977 **	C	Intron variant	1.737	0.0546	0.0531	376	0.1531	0.4978
** rs2074452 **	T	TF binding site ^e^	0.5028	0.0393	0.0465	429	0.1377	0.4478
** rs3826961 **	T	Intron variant	0.3317	0.0049	0.0037	5721	0.0339	0.1103
** rs3746162 **	T	Intron variant	0.3169	0.0035	0.0024	8716	0.0339	0.1103

^a^ Odds Ratio (OR). ^b^ *p*-value < 0.05. ^c^ False discovery rate_Benjamini–Hochberg procedure (FDR_BH). ^d^ False discovery rate_Benjamini–Yekutieli procedure (FDR¬_BY). ^e^ Transcription Factor (TF).

## Data Availability

Data are contained within the article and Supplementary Materials.

## Supplementary Materials

The following supporting information can be downloaded at: https://www.mdpi.com/article/10.3390/antiox11040721/s1, Supplemental Table S1—Amplicon and region of Chr19 covered by primers in this study. Also shown are sequencing metrics (i.e., run reports) for DNA sequencing.
