# Supplementary material for: A Genotype–Phenotype Analysis of Glutathione Peroxidase 4 in Human Atrial Myocardium and Its Association with Postoperative Atrial Fibrillation
Source: Antioxidants (Basel). 2022 Apr 6;11(4):721. doi: 10.3390/antiox11040721 (PMC9026099; doi:10.3390/antiox11040721)
Supplement: Supplementary file 1 [file antioxidants-11-00721-s001.zip › antioxidants-1650967-supplementary.pdf]

Table S1. Amplicons and regions of Chr19 covered by sequencing runs.

Chromosome	Amplicon_begin	Amplicon_end	Amplicon_Length
chr19	1101928	1102216	288
chr19	1103665	1103992	327
chr19	1104011	1104286	275
chr19	1104587	1104881	294
chr19	1104659	1104950	291
chr19	1105070	1105399	329
chr19	1105273	1105418	145
chr19	1105464	1105797	333
chr19	1105783	1106062	279
chr19	1106082	1106373	291
chr19	1106235	1106563	328
chr19	1106552	1106749	197
chr19	1106765	1107039	274
chr19	1107058	1107247	189
chr19	1113939	1114260	321

Run Summary

3.78 G	109
Total Bases	Key Signal

94%
ISP Loading

ISP Density

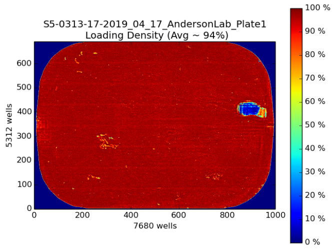

15,009,763
Total Reads

42%
Usable Reads

ISP Summary

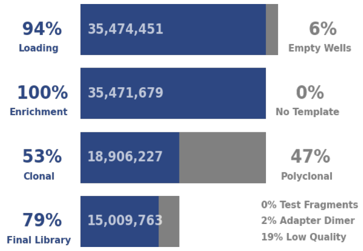

252 bp	278 bp	295 bp
Mean	Median	Mode

Read Length

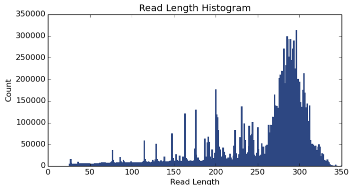

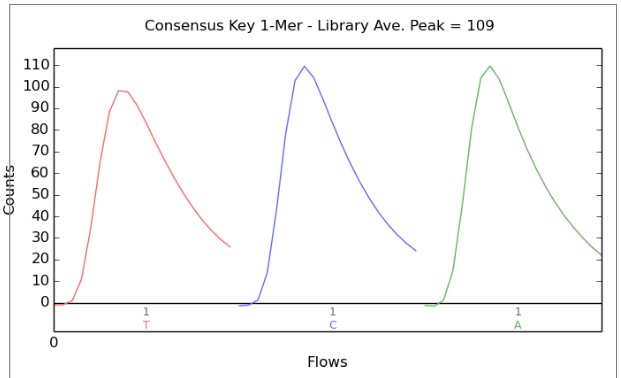

**Addressable Wells**	**37,849,615**	
With ISPs	35,474,451	93.7%
Live	35,471,679	100.0%
Test Fragment	110,607	00.3%
Library	35,361,072	99.7%

**Library ISPs**	**35,361,072**	
Filtered: Polyclonal	16,565,452	46.8%
Filtered: Low Quality	3,472,699	09.8%
Filtered: Adapter Dimer	313,158	00.9%
**Final Library ISPs**	**15,009,763**	**42.4%**

Barcode Name	Sample	Bases	$\geq Q20$	Reads	Mean Read Length	Read Length Histogram
No barcode	none	75,568,309	67,295,222	296,537	254 bp	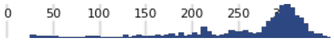
IonXpress_001	Sample 1	112,667,985	102,532,458	449,811	250 bp	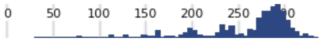
IonXpress_002	Sample 2	96,734,944	88,185,936	398,995	242 bp	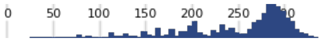
IonXpress_003	Sample 3	26,753,870	24,402,209	104,917	255 bp	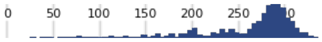
IonXpress_004	Sample 4	121,141,802	110,277,323	480,120	252 bp	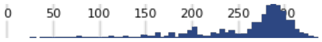
IonXpress_005	Sample 5	22,621,323	20,555,838	87,264	259 bp	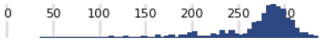
IonXpress_006	Sample 6	185,419,684	168,150,868	720,303	257 bp	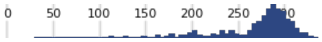
IonXpress_007	Sample 7	38,343,633	34,841,409	146,405	261 bp	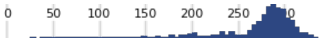

iontorrent by Thermo Fisher Scientific

IonXpress_008	Sample 8	62,777,065	56,968,394	247,434	253 bp	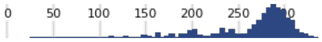
IonXpress_009	Sample 9	7,412,042	6,733,122	30,873	240 bp	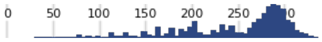
IonXpress_010	Sample 10	94,496,322	85,408,074	373,663	252 bp	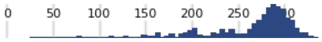
IonXpress_011	Sample 11	55,785,329	50,318,456	248,450	224 bp	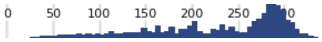
IonXpress_012	Sample 12	85,651,021	77,936,316	337,758	253 bp	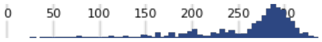
IonXpress_013	Sample 13	144,353,044	131,315,147	570,246	253 bp	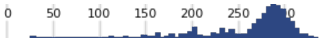
IonXpress_014	Sample 14	42,637,193	38,814,922	175,522	242 bp	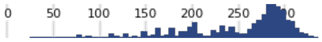
IonXpress_015	Sample 15	1,453,243	1,319,669	5,672	256 bp	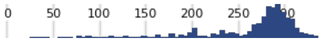
IonXpress_016	Sample 16	16,327,781	14,799,965	71,470	228 bp	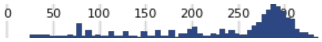
IonXpress_017	Sample 17	165,048,462	149,778,634	650,923	253 bp	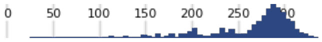
IonXpress_018	Sample 18	60,430,699	54,385,253	252,843	239 bp	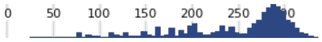
IonXpress_019	Sample 19	86,215,896	78,599,453	341,135	252 bp	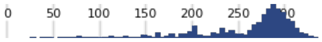
IonXpress_020	Sample 20	14,459,878	13,150,626	58,275	248 bp	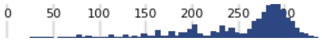
IonXpress_021	Sample 21	54,296,897	49,149,331	212,594	255 bp	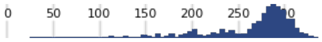
IonXpress_022	Sample 22	17,777,542	16,159,560	72,367	245 bp	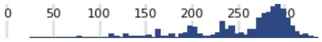
IonXpress_023	Sample 23	6,879,212	6,231,476	28,069	245 bp	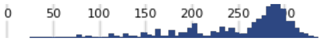
IonXpress_024	Sample 24	18,155,074	16,485,389	74,451	243 bp	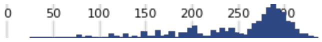
IonXpress_025	Sample 25	37,869,998	33,792,586	157,374	240 bp	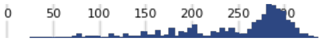
IonXpress_026	Sample 26	4,924,619	4,467,663	20,321	242 bp	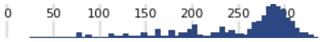
IonXpress_027	Sample 27	15,104,674	13,770,254	59,837	252 bp	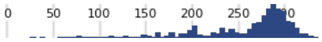
IonXpress_028	Sample 28	1,894,871	1,724,600	7,661	247 bp	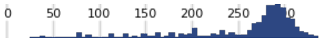
IonXpress_029	Sample 29	101,778,284	92,056,874	392,719	259 bp	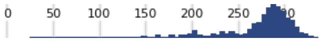
IonXpress_030	Sample 30	2,265,422	2,045,709	9,335	242 bp	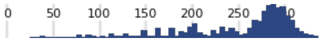
IonXpress_031	Sample 31	8,833,673	7,973,201	35,491	248 bp	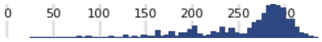
IonXpress_032	Sample 32	17,006,621	15,449,115	68,159	249 bp	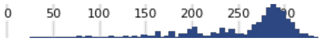
IonXpress_033	Sample 33	72,751,521	66,114,229	284,074	256 bp	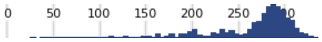

iontorrent
by Thermo Fisher Scientific

IonXpress_034	Sample 34	15,370,022	13,949,530	61,481	249 bp	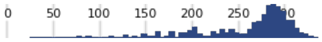
IonXpress_035	Sample 35	60,239,891	54,581,574	236,602	254 bp	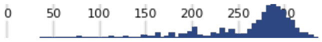
IonXpress_036	Sample 36	39,851,949	36,355,167	160,184	248 bp	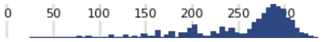
IonXpress_037	Sample 37	113,007,422	102,276,890	441,734	255 bp	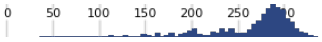
IonXpress_038	Sample 38	3,003,433	2,733,505	12,566	239 bp	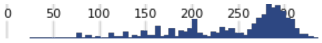
IonXpress_039	Sample 39	9,840	9,058	38	258 bp	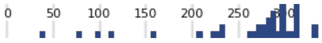
IonXpress_040	Sample 40	6,305,272	5,712,687	26,727	235 bp	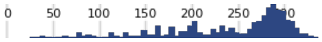
IonXpress_041	Sample 41	10,833,279	9,821,764	43,262	250 bp	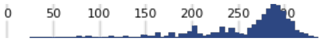
IonXpress_042	Sample 42	3,324,445	3,023,012	14,067	236 bp	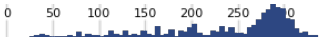
IonXpress_043	Sample 43	58,669,658	52,947,247	235,467	249 bp	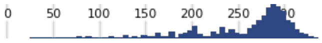
IonXpress_044	Sample 44	7,038,487	6,397,790	28,014	251 bp	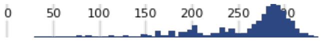
IonXpress_045	Sample 45	18,560,227	16,775,569	74,305	249 bp	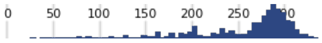
IonXpress_046	Sample 46	9,045,798	8,211,511	36,996	244 bp	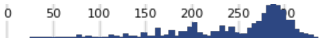
IonXpress_047	Sample 47	48,740,848	44,382,120	192,992	252 bp	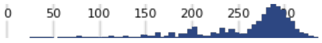
IonXpress_048	Sample 48	103,172,359	93,935,068	407,506	253 bp	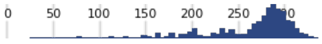
IonXpress_049	Sample 49	4,951,356	4,494,944	20,075	246 bp	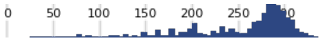
IonXpress_050	Sample 50	2,083,948	1,889,885	8,601	242 bp	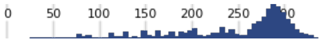
IonXpress_051	Sample 51	1,879,239	1,693,514	7,259	258 bp	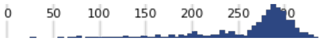
IonXpress_052	Sample 52	3,867,356	3,472,504	15,739	245 bp	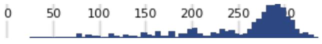
IonXpress_053	Sample 53	12,748,748	11,520,896	50,812	250 bp	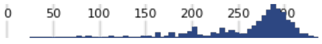
IonXpress_054	Sample 54	5,719,201	5,153,599	23,763	240 bp	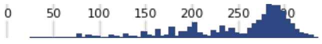
IonXpress_055	Sample 55	7,739,094	7,006,965	31,719	243 bp	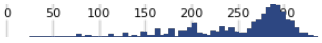
IonXpress_056	Sample 56	1,480,690	1,344,087	6,198	238 bp	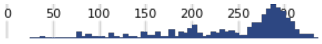
IonXpress_057	Sample 57	12,594,062	11,374,245	52,050	241 bp	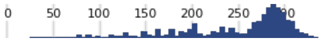
IonXpress_058	Sample 58	744,967	673,521	3,145	236 bp	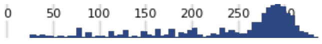
IonXpress_059	Sample 59	50,205,353	45,397,273	199,604	251 bp	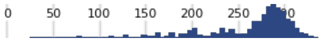

iontorrent
by Thermo Fisher Scientific

IonXpress_060	Sample 60	19,366,578	17,560,384	76,970	251 bp	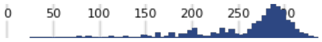
IonXpress_061	Sample 61	134,189,795	121,582,781	530,357	253 bp	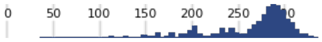
IonXpress_062	Sample 62	2,951,106	2,654,261	12,645	233 bp	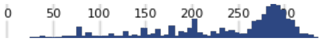
IonXpress_063	Sample 63	35,780,846	32,173,868	142,844	250 bp	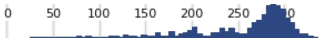
IonXpress_064	Sample 64	3,271,393	2,956,102	13,461	243 bp	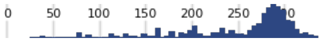
IonXpress_065	Sample 65	14,564,817	13,102,959	60,596	240 bp	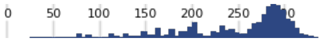
IonXpress_066	Sample 66	3,348,031	2,989,251	13,503	247 bp	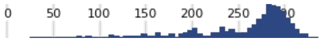
IonXpress_067	Sample 67	47,713,582	43,222,325	186,088	256 bp	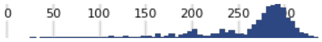
IonXpress_068	Sample 68	8,632,063	7,878,189	35,843	240 bp	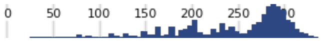
IonXpress_069	Sample 69	14,901,659	13,537,248	57,518	259 bp	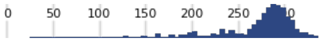
IonXpress_070	Sample 70	4,923,675	4,397,812	20,226	243 bp	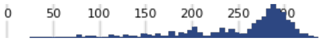
IonXpress_071	Sample 71	21,048,878	19,127,828	83,473	252 bp	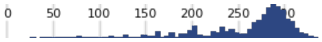
IonXpress_072	Sample 72	30,642,338	27,805,351	119,662	256 bp	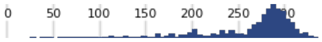
IonXpress_073	Sample 73	226,166,977	203,884,307	883,531	255 bp	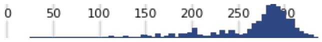
IonXpress_074	Sample 74	2,392,634	2,169,880	9,744	245 bp	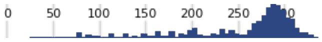
IonXpress_075	Sample 75	25,517,299	23,204,808	101,686	250 bp	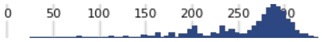
IonXpress_076	Sample 76	2,541,870	2,303,937	10,501	242 bp	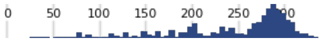
IonXpress_077	Sample 77	80,803,422	72,143,577	319,615	252 bp	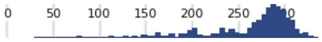
IonXpress_078	Sample 78	46,441,252	42,115,019	185,907	249 bp	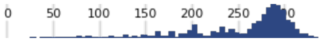
IonXpress_079	Sample 79	72,225,287	63,912,457	283,939	254 bp	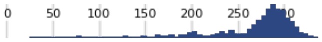
IonXpress_080	Sample 80	4,898,668	4,453,147	19,580	250 bp	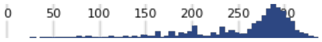
IonXpress_081	Sample 81	26,075,688	23,186,657	103,494	251 bp	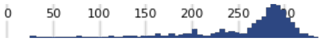
IonXpress_082	Sample 82	22,886,583	20,750,941	92,564	247 bp	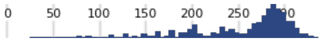
IonXpress_083	Sample 83	839,939	760,230	3,566	235 bp	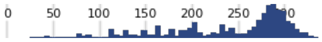
IonXpress_084	Sample 84	78,586,987	70,966,263	311,209	252 bp	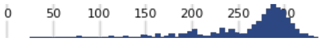
IonXpress_085	Sample 85	151,925,269	137,008,786	580,881	261 bp	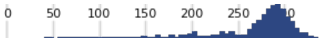

iontorrent
by Thermo Fisher Scientific

IonXpress_086	Sample 86	14,935,363	13,492,473	60,582	246 bp	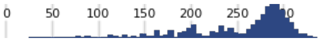
IonXpress_087	Sample 87	1,610,725	1,456,055	8,159	197 bp	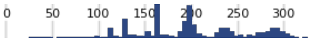
IonXpress_088	Sample 88	8,463,945	7,612,517	34,803	243 bp	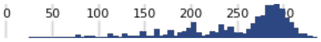
IonXpress_089	Sample 89	90,649,768	81,809,378	350,847	258 bp	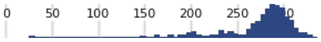
IonXpress_090	Sample 90	423,646	380,737	1,668	253 bp	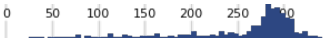
IonXpress_091	Sample 91	23,018,123	20,913,407	87,186	264 bp	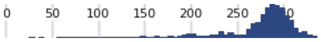
IonXpress_092	Sample 92	2,704,918	2,450,213	13,489	200 bp	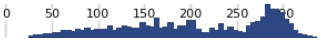
IonXpress_093	Sample 93	20,850,817	18,754,519	89,102	234 bp	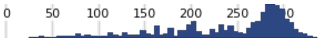
IonXpress_094	Sample 94	7,712,122	7,026,312	30,418	253 bp	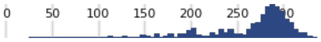
IonXpress_095	Sample 95	42,163,861	38,106,465	163,085	258 bp	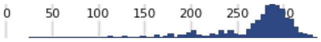
IonXpress_096	Sample 96	11,960,034	10,877,321	49,047	243 bp	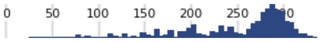

Test Fragment	Reads	Percent 50AQ17	Read Length Histogram
**TF_1**	**20,414**	**92**	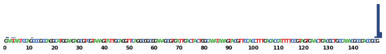

iontorrent by Thermo Fisher Scientific

Alignment Summary *(aligned to Homo sapiens)*

3.69 G
Total Alignment Bases

1.2X
Average Coverage Depth of Reference

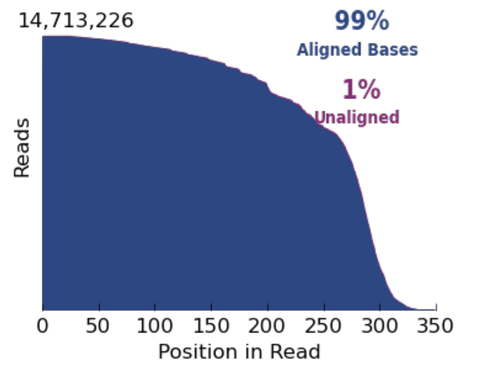

	Count	%
**Total Reads**	14,713,226	–
**Aligned Reads**	14,687,226	99.8%
**Unaligned Reads**	26,000	0.2%

99.3%
Mean Raw Accuracy 1x

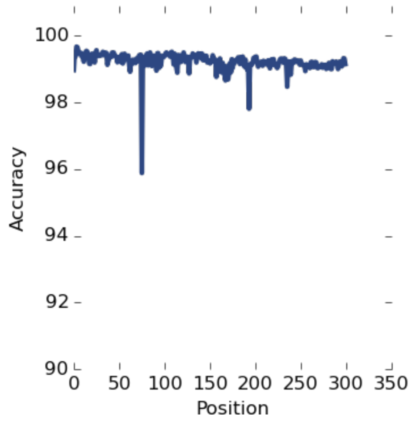

Alignment Quality

	AQ17	AQ20	Perfect
**Total Number of Bases [Mbp]**	3.53 G	3.24 G	2.2 G
**Mean Length [bp]**	246	231	166
**Longest Alignment [bp]**	356	356	345
**Mean Coverage Depth**	1.1	1.0	0.7

iontorrent by Thermo Fisher Scientific

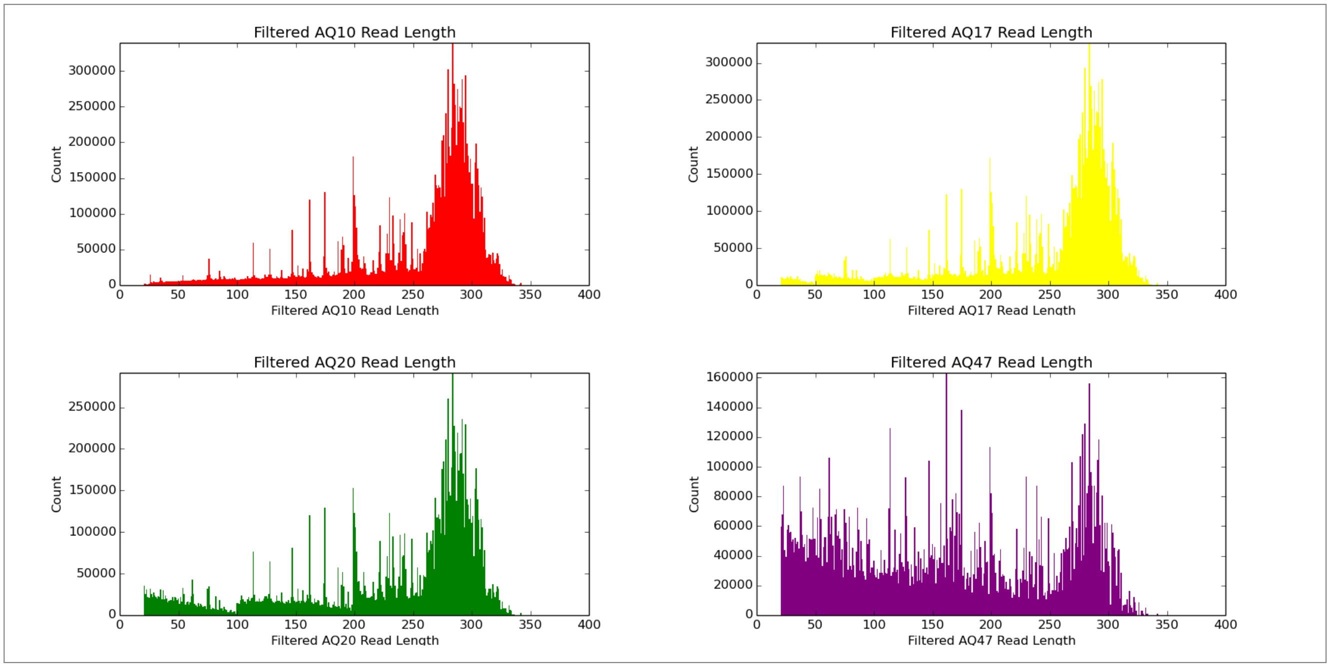

iontorrent by Thermo Fisher Scientific

coverageAnalysis

Library type: AmpliSeq DNA
Target regions: IAD164741_197_Designed

Barcode Name	Sample	Mapped Reads	On Target	Mean Depth	Uniformity
IonXpress_001	Sample 1	448,999	99.65%	1,693	52.18%
IonXpress_002	Sample 2	398,267	99.52%	1,449	93.04%
IonXpress_003	Sample 3	104,743	99.77%	402	93.65%
IonXpress_004	Sample 4	479,277	99.45%	1,815	93.81%
IonXpress_005	Sample 5	87,143	98.31%	336.3	95.16%
IonXpress_006	Sample 6	719,128	99.35%	2,775	94.63%
IonXpress_007	Sample 7	146,126	99.43%	574.4	94.99%
IonXpress_008	Sample 8	246,915	99.24%	938.6	93.78%
IonXpress_009	Sample 9	30,820	99.11%	110.7	92.61%
IonXpress_010	Sample 10	372,885	99.68%	1,418	94.03%
IonXpress_011	Sample 11	247,864	78.85%	716.1	91.39%
IonXpress_012	Sample 12	337,186	99.29%	1,281	94.07%
IonXpress_013	Sample 13	568,965	99.55%	2,163	93.49%
IonXpress_014	Sample 14	175,221	99.52%	639.2	93.27%
IonXpress_015	Sample 15	5,658	99.72%	21.8	94.03%
IonXpress_016	Sample 16	71,078	97.60%	242.7	94.57%
IonXpress_017	Sample 17	649,899	99.68%	2,477	93.26%
IonXpress_018	Sample 18	252,411	99.18%	902.4	93.95%
IonXpress_019	Sample 19	340,615	99.48%	1,292	93.68%
IonXpress_020	Sample 20	58,187	99.40%	216.4	93.42%
IonXpress_021	Sample 21	212,201	99.50%	812.8	93.71%
IonXpress_022	Sample 22	72,236	99.50%	266.4	82.40%
IonXpress_023	Sample 23	28,022	99.08%	102.7	93.79%
IonXpress_024	Sample 24	74,315	99.33%	271.6	93.61%
IonXpress_025	Sample 25	157,073	99.52%	566.6	92.67%
IonXpress_026	Sample 26	20,278	99.48%	73.74	93.88%
IonXpress_027	Sample 27	59,752	99.70%	226.8	93.31%
IonXpress_028	Sample 28	7,640	99.07%	28.33	95.16%
IonXpress_029	Sample 29	392,032	99.54%	1,525	94.26%
IonXpress_030	Sample 30	9,316	99.23%	33.84	92.23%
IonXpress_031	Sample 31	35,392	99.08%	132	[illegible]
IonXpress_032	Sample 32	68,029	99.37%	254.6	[illegible]

iontorrent by Thermo Fisher Scientific

IonXpress_032	Sample 32	[illegible]	[illegible]	[illegible]	[illegible]
IonXpress_033	Sample 33	283,601	99.49%	1,090	94.17%
IonXpress_034	Sample 34	61,371	99.44%	230.1	93.86%
IonXpress_035	Sample 35	236,309	99.48%	902.3	93.96%
IonXpress_036	Sample 36	159,934	99.35%	596.7	93.74%
IonXpress_037	Sample 37	441,159	99.67%	1,695	93.63%
IonXpress_038	Sample 38	12,548	99.36%	45.02	92.61%
IonXpress_039	Sample 39	38	100.00%	0.147	97.46%
IonXpress_040	Sample 40	26,660	99.28%	94.33	92.71%
IonXpress_041	Sample 41	43,186	99.49%	162.2	93.28%
IonXpress_042	Sample 42	14,033	97.50%	49.37	92.83%
IonXpress_043	Sample 43	235,055	99.41%	877.9	92.78%
IonXpress_044	Sample 44	27,979	99.21%	105.2	93.13%
IonXpress_045	Sample 45	74,196	99.42%	277.8	93.65%
IonXpress_046	Sample 46	36,947	99.55%	135.5	93.05%
IonXpress_047	Sample 47	192,708	99.47%	730.1	93.89%
IonXpress_048	Sample 48	406,803	99.41%	1,545	94.79%
IonXpress_049	Sample 49	20,042	99.49%	74.17	92.77%
IonXpress_050	Sample 50	8,583	99.55%	31.24	94.03%
IonXpress_051	Sample 51	7,251	99.74%	28.2	94.84%
IonXpress_052	Sample 52	15,705	99.48%	57.87	93.47%
IonXpress_053	Sample 53	50,723	99.50%	190.9	93.58%
IonXpress_054	Sample 54	23,733	99.22%	85.52	92.25%
IonXpress_055	Sample 55	31,661	99.31%	115.7	94.11%
IonXpress_056	Sample 56	6,188	99.03%	22.09	93.06%
IonXpress_057	Sample 57	51,960	99.27%	188.1	93.83%
IonXpress_058	Sample 58	3,129	98.95%	11.12	92.69%
IonXpress_059	Sample 59	199,188	99.70%	753.3	94.01%
IonXpress_060	Sample 60	76,808	99.10%	289.7	94.36%
IonXpress_061	Sample 61	529,631	99.62%	2,012	93.06%
IonXpress_062	Sample 62	12,620	99.35%	44.12	93.42%
IonXpress_063	Sample 63	142,635	99.65%	536.3	92.99%
IonXpress_064	Sample 64	13,435	99.27%	48.95	94.68%
IonXpress_065	Sample 65	60,481	99.43%	217.9	93.55%
IonXpress_066	Sample 66	13,471	99.45%	50.03	92.05%

IonXpress_067	Sample 67	185,789	99.53%	715	93.13%
IonXpress_068	Sample 68	35,778	99.64%	129.6	91.84%
IonXpress_069	Sample 69	57,393	99.38%	223.2	94.01%
IonXpress_070	Sample 70	20,191	99.12%	73.37	92.60%
IonXpress_071	Sample 71	83,355	99.55%	315.6	93.22%
IonXpress_072	Sample 72	119,449	99.46%	459.1	94.12%
IonXpress_073	Sample 73	882,042	99.65%	3,391	94.23%
IonXpress_074	Sample 74	9,726	99.34%	35.79	94.68%
IonXpress_075	Sample 75	101,512	99.73%	383.4	90.82%
IonXpress_076	Sample 76	10,480	99.57%	38.12	92.21%
IonXpress_077	Sample 77	319,103	99.58%	1,209	93.67%
IonXpress_078	Sample 78	185,673	99.52%	695.5	92.84%
IonXpress_079	Sample 79	283,241	99.46%	1,079	93.47%
IonXpress_080	Sample 80	19,546	99.33%	73.32	93.09%
IonXpress_081	Sample 81	103,255	99.51%	389.6	92.58%
IonXpress_082	Sample 82	92,404	99.75%	343.7	93.67%
IonXpress_083	Sample 83	3,554	99.30%	12.56	90.60%
IonXpress_084	Sample 84	310,609	99.37%	1,176	93.05%
IonXpress_085	Sample 85	580,128	99.65%	2,278	94.35%
IonXpress_086	Sample 86	60,460	99.45%	223.6	92.37%
IonXpress_087	Sample 87	8,146	99.66%	24.12	43.50%
IonXpress_088	Sample 88	34,735	99.32%	126.4	92.03%
IonXpress_089	Sample 89	350,013	99.52%	1,358	94.08%
IonXpress_090	Sample 90	1,665	98.86%	6.329	93.51%
IonXpress_091	Sample 91	87,057	99.48%	345	92.97%
IonXpress_092	Sample 92	13,464	59.05%	28.57	87.72%
IonXpress_093	Sample 93	88,916	92.91%	298.4	90.43%
IonXpress_094	Sample 94	30,346	99.51%	115.6	93.41%
IonXpress_095	Sample 95	162,802	99.49%	631.5	94.12%
IonXpress_096	Sample 96	48,950	99.08%	178.7	92.40%

1 5 items per page 1 - 96 of 96 items

iontorrent by Thermo Fisher Scientific

Analysis Details

**Run Name**	R_2019_04_18_08_31_26_user_S5-0313-17-2019_04_17_AndersonLab_Plate1
**Run Date**	April 18, 2019, 8:33 a.m.
**Run Flows**	500
**Projects**	Anderson_Lab
**Sample**	Sample_2, Sample_1, Sample_15, Sample_10, Sample_11, Sample_13, Sample_14, Sample_16, Sample_8, Sample_9, Sample_6, Sample_7, Sample_4, Sample_5, Sample_3, Sample_12, Sample_50, Sample_51, Sample_52, Sample_53, Sample_54, Sample_55, Sample_56, Sample_57, Sample_58, Sample_59, Sample_25, Sample_24, Sample_27, Sample_26, Sample_21, Sample_20, Sample_23, Sample_22, Sample_29, Sample_28, Sample_36, Sample_37, Sample_34, Sample_35, Sample_32, Sample_33, Sample_30, Sample_31, Sample_38, Sample_39, Sample_83, Sample_82, Sample_81, Sample_80, Sample_87, Sample_86, Sample_85, Sample_84, Sample_89, Sample_88, Sample_94, Sample_95, Sample_96, Sample_90, Sample_91, Sample_92, Sample_93, Sample_18, Sample_19, Sample_17, Sample_69, Sample_68, Sample_61, Sample_60, Sample_63, Sample_62, Sample_65, Sample_64, Sample_67, Sample_66, Sample_78, Sample_79, Sample_72, Sample_73, Sample_70, Sample_71, Sample_76, Sample_77, Sample_74, Sample_75, Sample_49, Sample_48, Sample_47, Sample_46, Sample_45, Sample_44, Sample_43, Sample_42, Sample_41, Sample_40
**Reference**	
**Instrument**	S5-0313
**Operation Mode**	Customer mode
**Flow Order**	TACGTACGTCTGAGCATCGATCGATGTACAGC
**Library Key**	TCAG
**TF Key**	ATCG
**Chip Barcode**	DAEI01956
**Chip Check**	Passed
**Chip Type**	530
**Chip Data**	tiled
**Chip Lot Number**	QVB555
**Chip Wafer**	16
**Barcode Set**	IonXpress
**Analysis Name**	Auto_user_S5-0313-17-2019_04_17_AndersonLab_Plate1_168
**Analysis Date**	April 18, 2019, 7:09 p.m.
**Analysis Flows**	0
**runID**	16MFT
**BeadFind Args**	justBeadFind –args-json /opt/ion/config/args_530_beadfind.json
**Analysis Args**	Analysis –args-json /opt/ion/config/args_530_analysis.json
**Pre-BaseCaller**	BaseCaller –barcode-filter-minreads 10 –phasing-residual-filter=2.0 –wells-normalization on
**Calibration Args**	Calibration –num-calibration-regions 1,1
**BaseCaller Args**	BaseCaller –barcode-filter-minreads 10 –phasing-residual-filter=2.0 –num-unfiltered 1000 –barcode-filter-postpone 1 –qual-filter true –qual-filter-slope 0.040 –qual-filter-offset 1.0 –wells-normalization on
**Alignment Args**	tmap mapall -q 50000 ... stage1 map4
**IonStats Args**	ionstats alignment
**Analysis Parameters**	default

Chef Summary

Chef Template Prep Information:

**Chef Last Updated**	April 18, 2019, 8:29 a.m.
**Chef Instrument Name**	242470708
**Chef Operation Mode**	Customer Mode
**Sample Position**	1
**Tip Rack Barcode**	48726009E
**Chip Type 1**	530v1
**Chip Type 2**	530v1
**Chip Expiration 1**	None
**Chip Expiration 2**	None
**Templating Kit Type**	Ion 510
amp; Ion 520	amp; Ion 530 Kit-Chef
**Chef Flexible Workflow**	
**Reagent Expiration**	191130
**Reagent Lot Number**	2012420
**Reagent Part Number**	A34018C
**Reagent Cartridge Serial Number**	None
**Solution Lot Number**	2049981
**Solution Part Number**	A27754C
**Templating Protocol Planned**	(use instrument default)
**Solution Cartridge Serial Number**	None
**Solution Expiration**	191231
**Templating Protocol Executed**	(use instrument default)
**Chef Script Version**	803
**Chef Package Version**	IC.5.10.0
**Start Time**	April 17, 2019, 3:47 p.m.
**Completion Time**	April 18, 2019, 8:29 a.m.

S5 Consumables Summary

**Chip Type**	530v1
**Chip Barcode**	DAEI01956

Product Description	Part Number	Lot Number	Exp. Date	Remaining Uses
Ion S5 Cleaning Solution	100031096	1977166	2019/05/31	2
Ion S5 Sequencing Reagent	INS1012841B	2031538	2019/10/31	1
Ion S5 Wash Solution	100031091B	2033804	2019/10/31	1

iontorrent
by Thermo Fisher Scientific

Software Version

**Torrent_Suite**	5.10.1
**host**	tsvm
**ion-analysis**	5.10.11-1
**ion-dbreports**	5.10.27-1
**ion-gpu**	5.10.0-1
**ion-pipeline**	5.10.10-1
**ion-torrentpy**	5.10.9-1
**ion-torrentr**	5.10.10-1
**S5 Script**	0.1.31
**LiveView**	2389
**DataCollect**	3606
**OIA**	51000
**OS**	35
**Graphics**	97
**Ion_Chef**	IC.5.10.0

Run Summary

2.88 G	84
Total Bases	Key Signal

93%
ISP Loading

ISP Density

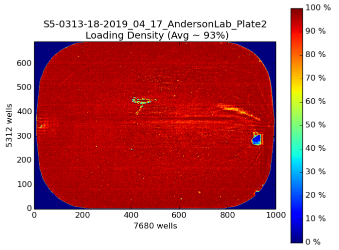

11,521,921
Total Reads

33%
Usable Reads

ISP Summary

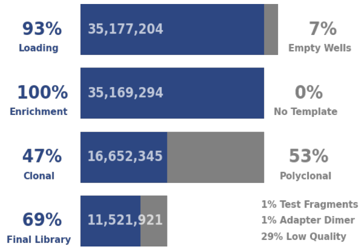

250 bp	277 bp	288 bp
Mean	Median	Mode

Read Length

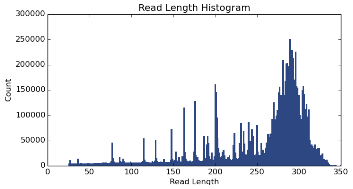

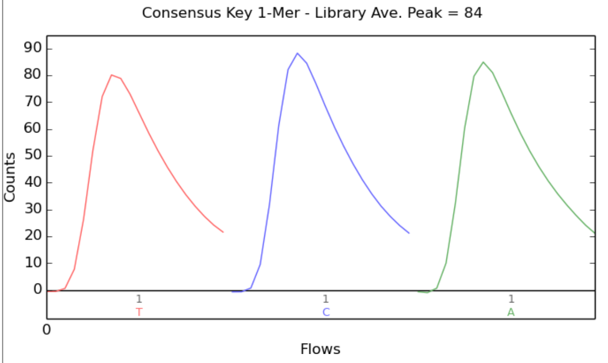

**Addressable Wells**	**37,849,615**	
With ISPs	35,177,204	92.9%
Live	35,169,294	100.0%
Test Fragment	204,196	00.6%
Library	34,965,098	99.4%

**Library ISPs**	**34,965,098**	
Filtered: Polyclonal	18,516,949	53.0%
Filtered: Low Quality	4,770,999	13.6%
Filtered: Adapter Dimer	155,229	00.4%
**Final Library ISPs**	**11,521,921**	**33.0%**

Barcode Name	Sample	Bases	$\geq Q20$	Reads	Mean Read Length	Read Length Histogram
No barcode	none	78,152,025	68,412,424	313,906	248 bp	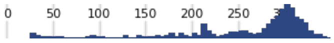
IonXpress_001	Sample 1	1,800,296	1,612,533	7,006	256 bp	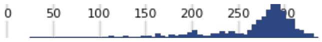
IonXpress_002	Sample 2	4,775,463	4,283,286	17,993	265 bp	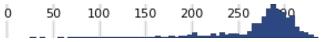
IonXpress_003	Sample 3	3,286,281	2,950,453	12,373	265 bp	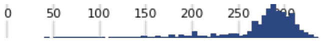
IonXpress_004	Sample 4	8,599,385	7,718,605	32,695	263 bp	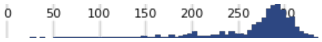
IonXpress_005	Sample 5	31,348,138	28,117,294	124,488	251 bp	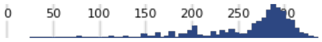
IonXpress_006	Sample 6	34,551,791	30,925,350	135,960	254 bp	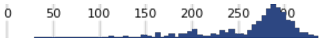
IonXpress_007	Sample 7	18,356,376	16,480,417	73,929	248 bp	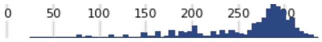

iontorrent by Thermo Fisher Scientific

IonXpress_008	Sample 8	42,126,634	37,707,322	168,050	250 bp	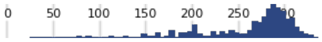
IonXpress_009	Sample 9	25,042,326	22,421,450	100,585	248 bp	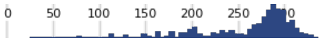
IonXpress_010	Sample 10	3,777,633	3,373,782	14,463	261 bp	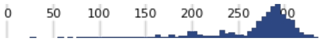
IonXpress_011	Sample 11	48,482,784	43,232,892	204,697	236 bp	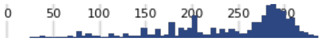
IonXpress_012	Sample 12	44,024,504	39,586,419	188,856	233 bp	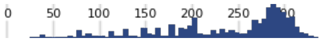
IonXpress_013	Sample 13	7,740,787	6,932,734	30,348	255 bp	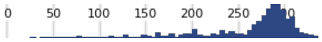
IonXpress_014	Sample 14	40,248,175	36,180,688	160,636	250 bp	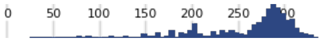
IonXpress_015	Sample 15	393,416	352,194	1,526	257 bp	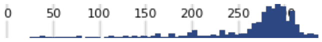
IonXpress_016	Sample 16	5,349,212	4,783,247	20,722	258 bp	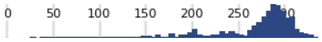
IonXpress_017	Sample 17	16,920,232	15,160,292	67,060	252 bp	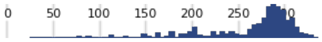
IonXpress_018	Sample 18	30,316,885	26,942,699	120,693	251 bp	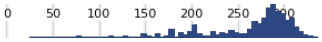
IonXpress_019	Sample 19	3,325,386	2,990,373	12,926	257 bp	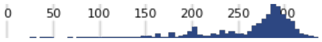
IonXpress_020	Sample 20	2,216,784	1,993,295	8,788	252 bp	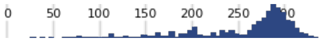
IonXpress_021	Sample 21	3,560,977	3,192,154	13,866	256 bp	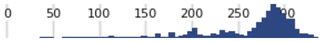
IonXpress_022	Sample 22	6,435,138	5,777,663	24,961	257 bp	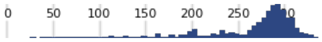
IonXpress_023	Sample 23	16,905,721	15,089,562	72,769	232 bp	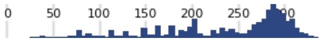
IonXpress_024	Sample 24	13,239,488	11,871,811	56,078	236 bp	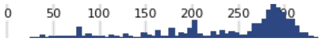
IonXpress_025	Sample 25	6,175,515	5,437,705	24,226	254 bp	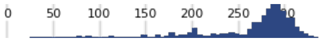
IonXpress_026	Sample 26	126,647,350	113,091,171	487,825	259 bp	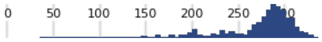
IonXpress_027	Sample 27	2,299,997	2,071,665	8,816	260 bp	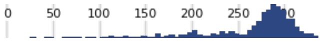
IonXpress_028	Sample 28	8,058,909	7,256,691	31,329	257 bp	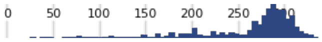
IonXpress_029	Sample 29	14,491,546	12,972,445	57,317	252 bp	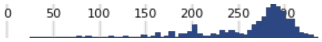
IonXpress_030	Sample 30	24,775,807	22,081,502	98,726	250 bp	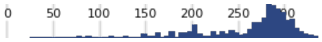
IonXpress_031	Sample 31	10,874,410	9,639,860	43,138	252 bp	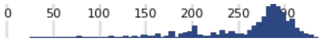
IonXpress_032	Sample 32	43,999,395	39,466,436	172,346	255 bp	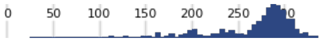
IonXpress_033	Sample 33	25,799,409	23,152,008	99,987	258 bp	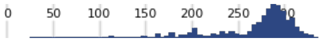

iontorrent
by Thermo Fisher Scientific

IonXpress_034	Sample 34	16,417,670	14,742,314	65,408	251 bp	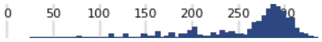
IonXpress_035	Sample 35	10,109,975	8,984,934	40,579	249 bp	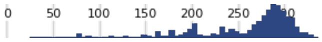
IonXpress_036	Sample 36	110,965,129	100,081,849	477,515	232 bp	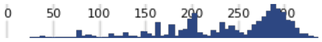
IonXpress_037	Sample 37	16,149,977	14,428,526	63,616	253 bp	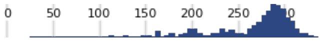
IonXpress_038	Sample 38	194,806,277	174,805,124	766,023	254 bp	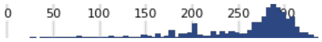
IonXpress_039	Sample 39	6,412,240	5,738,863	24,611	260 bp	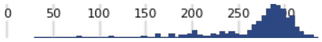
IonXpress_040	Sample 40	3,431,070	3,055,372	13,181	260 bp	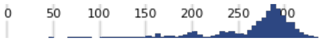
IonXpress_041	Sample 41	5,516,113	4,941,154	21,945	251 bp	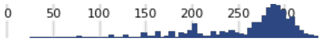
IonXpress_042	Sample 42	24,071,205	21,594,581	95,313	252 bp	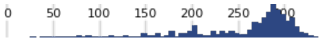
IonXpress_043	Sample 43	2,952,803	2,634,196	11,449	257 bp	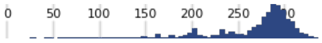
IonXpress_044	Sample 44	5,858,050	5,254,417	22,776	257 bp	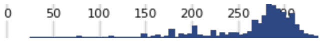
IonXpress_045	Sample 45	9,692,131	8,622,717	37,835	256 bp	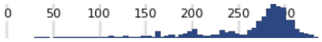
IonXpress_046	Sample 46	105,006,976	94,115,534	417,495	251 bp	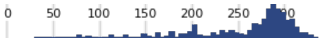
IonXpress_047	Sample 47	55,468,299	49,796,732	229,038	242 bp	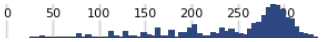
IonXpress_048	Sample 48	67,487,779	60,547,737	285,888	236 bp	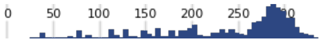
IonXpress_049	Sample 49	167,095,100	149,509,830	653,705	255 bp	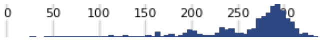
IonXpress_050	Sample 50	141,747,732	126,851,390	567,854	249 bp	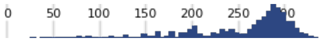
IonXpress_051	Sample 51	7,096,339	6,307,349	27,345	259 bp	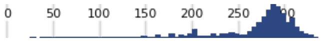
IonXpress_052	Sample 52	8,963,216	7,953,850	34,403	260 bp	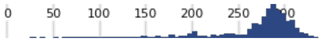
IonXpress_053	Sample 53	4,574,336	4,082,433	17,673	258 bp	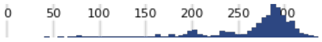
IonXpress_054	Sample 54	10,435,897	9,298,109	40,097	260 bp	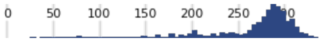
IonXpress_055	Sample 55	5,072,860	4,543,178	19,556	259 bp	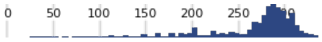
IonXpress_056	Sample 56	1,761,788	1,579,102	6,790	259 bp	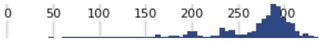
IonXpress_057	Sample 57	4,410,731	3,915,006	16,923	260 bp	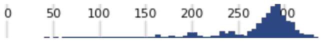
IonXpress_058	Sample 58	19,858,122	17,786,975	75,849	261 bp	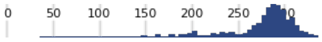
IonXpress_059	Sample 59	98,213,491	87,728,932	408,285	240 bp	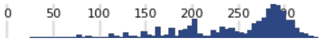

iontorrent
by Thermo Fisher Scientific

IonXpress_060	Sample 60	27,032,340	24,066,171	121,834	221 bp	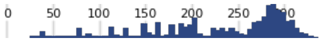
IonXpress_061	Sample 61	26,440,843	23,535,712	106,351	248 bp	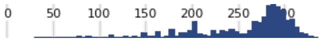
IonXpress_062	Sample 62	4,398,823	3,872,196	17,191	255 bp	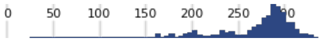
IonXpress_063	Sample 63	16,411,210	14,527,107	66,341	247 bp	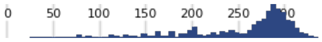
IonXpress_064	Sample 64	2,215,785	1,964,507	8,480	261 bp	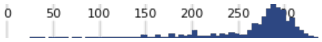
IonXpress_065	Sample 65	10,201,065	9,060,585	40,547	251 bp	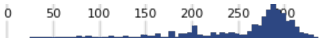
IonXpress_066	Sample 66	13,647,489	12,076,816	54,753	249 bp	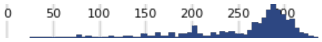
IonXpress_067	Sample 67	5,719,699	5,110,166	21,999	259 bp	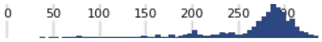
IonXpress_068	Sample 68	15,378,701	13,846,621	59,840	256 bp	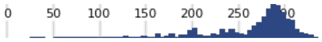
IonXpress_069	Sample 69	7,007,407	6,291,871	26,537	264 bp	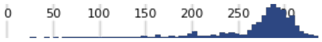
IonXpress_070	Sample 70	991,769	875,249	3,805	260 bp	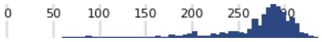
IonXpress_071	Sample 71	74,081,406	65,892,923	305,189	242 bp	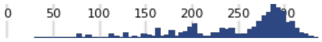
IonXpress_072	Sample 72	91,731,425	81,964,370	373,179	245 bp	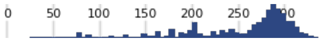
IonXpress_073	Sample 73	189,128,136	167,468,117	742,296	254 bp	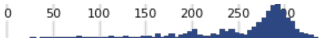
IonXpress_074	Sample 74	10,317,447	9,250,441	39,659	260 bp	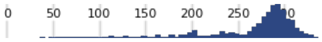
IonXpress_075	Sample 75	13,803,941	12,378,822	54,399	253 bp	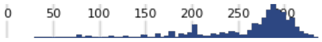
IonXpress_076	Sample 76	4,289,657	3,819,227	16,315	262 bp	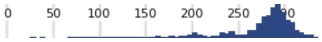
IonXpress_077	Sample 77	2,960,456	2,622,315	11,566	255 bp	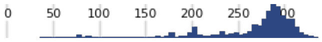
IonXpress_078	Sample 78	14,488,421	12,970,285	56,879	254 bp	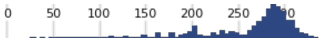
IonXpress_079	Sample 79	7,623,989	6,643,848	29,926	254 bp	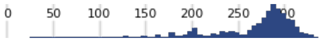
IonXpress_080	Sample 80	12,619,832	11,312,518	48,985	257 bp	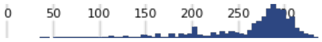
IonXpress_081	Sample 81	443,568	391,493	1,691	262 bp	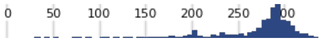
IonXpress_082	Sample 82	268,531	240,237	1,019	263 bp	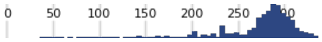
IonXpress_083	Sample 83	7,269,866	6,439,640	31,595	230 bp	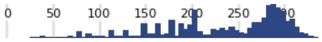
IonXpress_084	Sample 84	176,307,557	157,573,724	692,502	254 bp	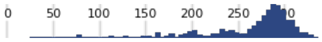
IonXpress_085	Sample 85	109,551,855	97,576,023	420,871	260 bp	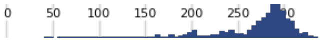

iontorrent
by Thermo Fisher Scientific

IonXpress_086	Sample 86	7,860	7,025	29	271 bp	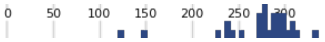
IonXpress_087	Sample 87	31,743,862	28,533,145	125,028	253 bp	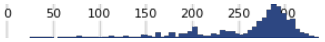
IonXpress_088	Sample 88	195,287	173,981	745	262 bp	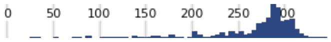
IonXpress_089	Sample 89	35,217,930	31,376,067	140,194	251 bp	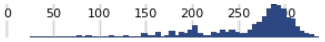
IonXpress_090	Sample 90	2,695,337	2,396,807	10,306	261 bp	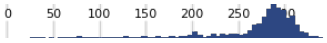
IonXpress_091	Sample 91	8,714,883	7,806,386	33,119	263 bp	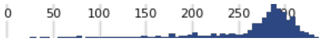
IonXpress_092	Sample 92	10,969,292	9,797,910	43,077	254 bp	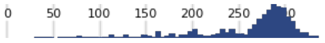
IonXpress_093	Sample 93	5,553,696	4,944,761	21,461	258 bp	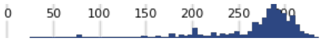
IonXpress_094	Sample 94	612,569	550,242	2,303	265 bp	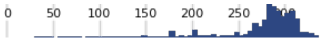
IonXpress_095	Sample 95	45,325,308	40,340,530	180,882	250 bp	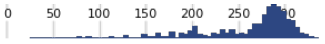
IonXpress_096	Sample 96	41,743,177	37,489,895	160,792	259 bp	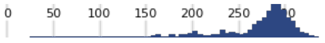

Test Fragment	Reads	Percent 50AQ17	Read Length Histogram
**TF_1**	**4,697**	**89**	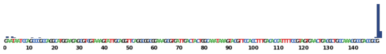

Alignment Summary *(aligned to Homo sapiens)*

**2.79 G**	**0.9X**
Total Alignment Bases	Average Coverage Depth of Reference

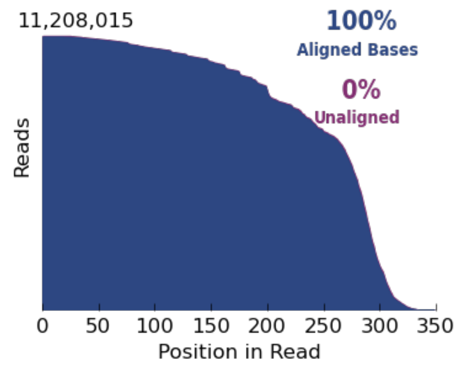

	Count	%
**Total Reads**	11,208,015	–
**Aligned Reads**	11,186,287	99.8%
**Unaligned Reads**	21,728	0.2%

99.3%
Mean Raw Accuracy 1x

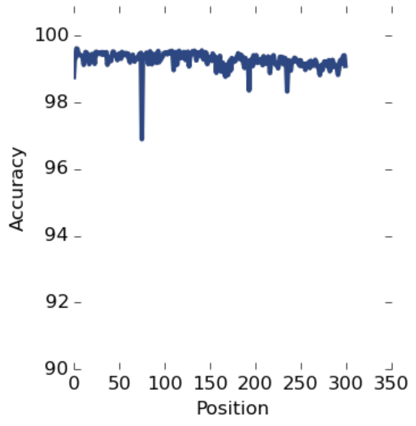

Alignment Quality

	AQ17	AQ20	Perfect
**Total Number of Bases [Mbp]**	2.67 G	2.48 G	1.76 G
**Mean Length [bp]**	246	234	174
**Longest Alignment [bp]**	352	352	345
**Mean Coverage Depth**	0.9	0.8	0.6

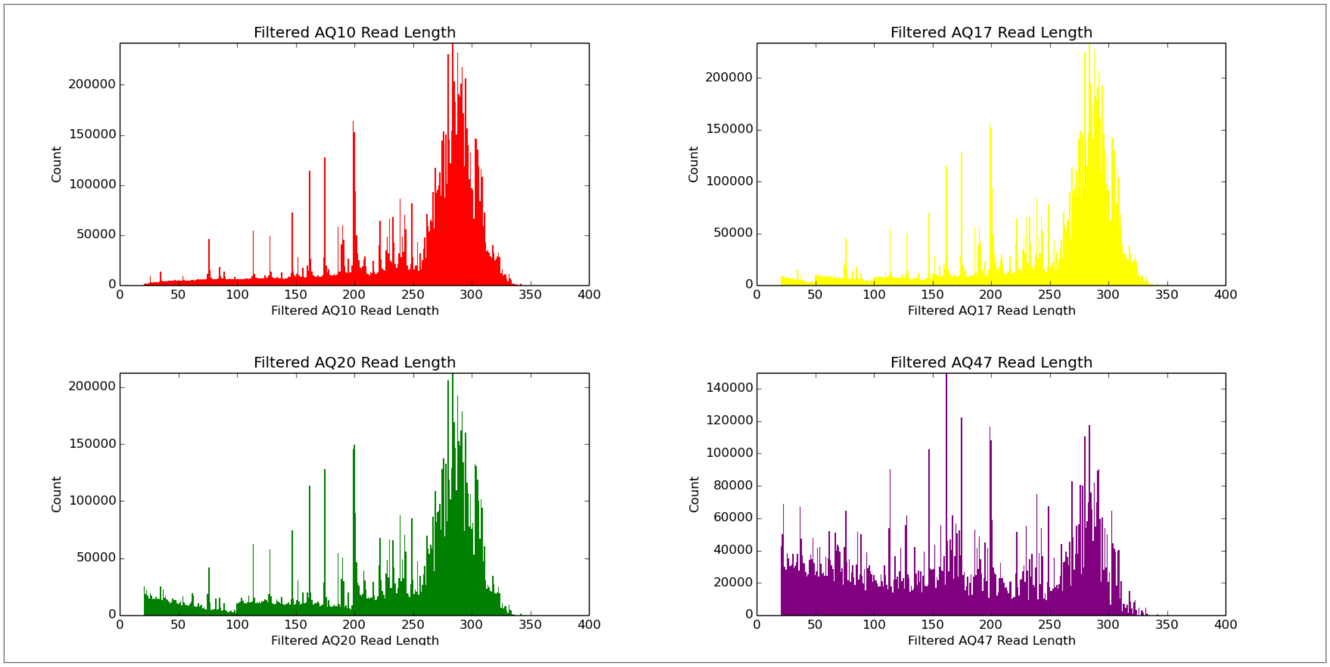
Filtered AQ10 Read Length
Count
Filtered AQ10 Read Length
Filtered AQ17 Read Length
Count
Filtered AQ17 Read Length
Filtered AQ20 Read Length
Count
Filtered AQ20 Read Length
Filtered AQ47 Read Length
Count
Filtered AQ47 Read Length

coverageAnalysis

Library type: AmpliSeq DNA
Target regions: IAD164741_197_Designed

Barcode Name	Sample	Mapped Reads	On Target	Mean Depth	Uniformity
IonXpress_001	Sample 1	6,990	98.90%	26.93	95.47%
IonXpress_002	Sample 2	17,961	99.49%	71.64	95.21%
IonXpress_003	Sample 3	12,353	99.20%	49.16	92.79%
IonXpress_004	Sample 4	32,637	99.35%	128.9	96.06%
IonXpress_005	Sample 5	124,245	99.14%	468.8	94.56%
IonXpress_006	Sample 6	135,665	99.24%	517.2	95.30%
IonXpress_007	Sample 7	73,756	99.08%	274.6	94.81%
IonXpress_008	Sample 8	167,652	99.19%	630	94.48%
IonXpress_009	Sample 9	100,366	99.27%	375	95.92%
IonXpress_010	Sample 10	14,445	99.54%	56.67	90.90%
IonXpress_011	Sample 11	204,197	99.13%	724.6	92.01%
IonXpress_012	Sample 12	188,406	98.65%	657.3	87.93%
IonXpress_013	Sample 13	30,287	99.29%	116	95.76%
IonXpress_014	Sample 14	160,304	99.16%	602.3	93.16%
IonXpress_015	Sample 15	1,522	99.21%	5.889	92.42%
IonXpress_016	Sample 16	20,668	99.41%	80.14	92.34%
IonXpress_017	Sample 17	66,929	99.13%	253.1	95.15%
IonXpress_018	Sample 18	120,518	99.23%	453.2	93.33%
IonXpress_019	Sample 19	12,898	99.32%	49.82	92.13%
IonXpress_020	Sample 20	8,780	99.34%	33.21	94.14%
IonXpress_021	Sample 21	13,842	99.42%	53.34	91.54%
IonXpress_022	Sample 22	24,912	99.55%	96.59	94.77%
IonXpress_023	Sample 23	72,600	99.03%	252.7	94.23%
IonXpress_024	Sample 24	55,924	97.18%	196.8	92.93%
IonXpress_025	Sample 25	24,174	99.33%	92.31	93.40%
IonXpress_026	Sample 26	486,994	99.32%	1,897	93.71%
IonXpress_027	Sample 27	8,801	99.24%	34.46	93.05%
IonXpress_028	Sample 28	31,265	99.17%	120.5	94.05%
IonXpress_029	Sample 29	57,187	99.32%	217	94.78%
IonXpress_030	Sample 30	98,522	99.37%	371.1	94.20%
IonXpress_031	Sample 31	43,068	99.47%	163	[illegible]
IonXpress_032	Sample 32	172,006	99.51%	660.3	95.83%

iontorrent by Thermo Fisher Scientific

IonXpress_032	Sample 32	[illegible]	[illegible]	[illegible]	[illegible]
IonXpress_033	Sample 33	99,801	99.54%	387	95.00%
IonXpress_034	Sample 34	65,283	99.52%	246.5	95.45%
IonXpress_035	Sample 35	40,511	99.44%	151.6	93.30%
IonXpress_036	Sample 36	476,645	98.56%	1,658	91.49%
IonXpress_037	Sample 37	63,515	99.46%	242.1	92.96%
IonXpress_038	Sample 38	764,685	99.37%	2,918	93.97%
IonXpress_039	Sample 39	24,563	99.48%	96.1	93.47%
IonXpress_040	Sample 40	13,163	99.57%	51.47	89.56%
IonXpress_041	Sample 41	21,901	99.25%	82.59	94.95%
IonXpress_042	Sample 42	95,167	99.39%	360.6	94.92%
IonXpress_043	Sample 43	11,424	98.83%	44.06	90.79%
IonXpress_044	Sample 44	22,739	99.27%	87.7	88.52%
IonXpress_045	Sample 45	37,778	99.63%	145.6	94.17%
IonXpress_046	Sample 46	416,799	99.48%	1,576	95.67%
IonXpress_047	Sample 47	228,587	99.37%	831.6	95.28%
IonXpress_048	Sample 48	285,063	99.14%	1,012	94.61%
IonXpress_049	Sample 49	652,546	99.45%	2,505	88.38%
IonXpress_050	Sample 50	566,888	99.20%	2,121	94.17%
IonXpress_051	Sample 51	27,291	99.52%	106.4	93.84%
IonXpress_052	Sample 52	34,347	99.49%	134.2	95.13%
IonXpress_053	Sample 53	17,648	99.59%	68.62	93.23%
IonXpress_054	Sample 54	40,040	99.61%	156.6	94.73%
IonXpress_055	Sample 55	19,515	99.49%	76.06	94.21%
IonXpress_056	Sample 56	6,777	99.66%	26.5	86.12%
IonXpress_057	Sample 57	16,891	99.66%	66.18	91.26%
IonXpress_058	Sample 58	75,715	99.53%	298.2	95.68%
IonXpress_059	Sample 59	407,447	99.51%	1,473	93.86%
IonXpress_060	Sample 60	121,369	96.34%	399.8	92.32%
IonXpress_061	Sample 61	106,193	99.27%	395.6	94.26%
IonXpress_062	Sample 62	17,161	99.39%	65.87	91.11%
IonXpress_063	Sample 63	66,228	99.28%	245.5	95.15%
IonXpress_064	Sample 64	8,460	99.28%	33.17	92.57%
IonXpress_065	Sample 65	40,469	99.26%	152.6	95.06%
IonXpress_066	Sample 66	54,642	99.28%	204.2	95.40%

iontorrent by Thermo Fisher Scientific

IonXpress_067	Sample 67	21,966	99.34%	85.64	95.04%
IonXpress_068	Sample 68	59,737	99.57%	230.9	92.78%
IonXpress_069	Sample 69	26,492	99.58%	105.2	96.02%
IonXpress_070	Sample 70	3,800	99.37%	14.84	91.53%
IonXpress_071	Sample 71	304,783	99.47%	1,111	93.84%
IonXpress_072	Sample 72	372,523	99.30%	1,373	93.08%
IonXpress_073	Sample 73	740,692	99.41%	2,834	93.27%
IonXpress_074	Sample 74	39,595	99.48%	154.7	93.53%
IonXpress_075	Sample 75	54,296	99.19%	206.4	92.30%
IonXpress_076	Sample 76	16,287	99.54%	64.34	87.53%
IonXpress_077	Sample 77	11,543	99.46%	44.35	88.44%
IonXpress_078	Sample 78	56,800	99.50%	217.3	95.49%
IonXpress_079	Sample 79	29,872	99.43%	114	92.47%
IonXpress_080	Sample 80	48,895	99.42%	189.1	94.80%
IonXpress_081	Sample 81	1,686	99.64%	6.654	83.73%
IonXpress_082	Sample 82	1,018	99.90%	4.042	89.58%
IonXpress_083	Sample 83	31,521	99.06%	108.5	90.61%
IonXpress_084	Sample 84	690,921	99.45%	2,646	93.98%
IonXpress_085	Sample 85	420,261	99.40%	1,642	92.68%
IonXpress_086	Sample 86	29	100.00%	0.117	97.91%
IonXpress_087	Sample 87	124,794	99.31%	475.4	93.59%
IonXpress_088	Sample 88	743	99.19%	2.908	86.32%
IonXpress_089	Sample 89	139,889	99.13%	526.5	95.28%
IonXpress_090	Sample 90	10,283	98.99%	40.21	94.17%
IonXpress_091	Sample 91	33,067	99.53%	130.7	94.60%
IonXpress_092	Sample 92	43,007	99.39%	164.4	95.02%
IonXpress_093	Sample 93	21,425	99.40%	83.42	85.59%
IonXpress_094	Sample 94	2,300	99.13%	9.153	79.45%
IonXpress_095	Sample 95	180,514	99.15%	677.4	93.59%
IonXpress_096	Sample 96	160,423	99.22%	625	90.90%

1 | 5 items per page | 1 - 96 of 96 items

iontorrent by Thermo Fisher Scientific

Analysis Details

**Run Name**	R_2019_04_18_11_10_38_user_S5-0313-18-2019_04_17_AndersonLab_Plate2
**Run Date**	April 18, 2019, 11:12 a.m.
**Run Flows**	500
**Projects**	Anderson_Lab
**Sample**	Sample_46, Sample_45, Sample_44, Sample_43, Sample_42, Sample_41, Sample_40, Sample_2, Sample_1, Sample_15, Sample_10, Sample_11, Sample_13, Sample_14, Sample_16, Sample_8, Sample_9, Sample_6, Sample_7, Sample_4, Sample_5, Sample_3, Sample_12, Sample_50, Sample_51, Sample_52, Sample_53, Sample_54, Sample_55, Sample_56, Sample_57, Sample_58, Sample_59, Sample_25, Sample_24, Sample_27, Sample_26, Sample_21, Sample_20, Sample_23, Sample_22, Sample_29, Sample_28, Sample_36, Sample_37, Sample_34, Sample_35, Sample_32, Sample_33, Sample_30, Sample_31, Sample_38, Sample_39, Sample_83, Sample_82, Sample_81, Sample_80, Sample_87, Sample_86, Sample_85, Sample_84, Sample_89, Sample_88, Sample_94, Sample_95, Sample_96, Sample_90, Sample_91, Sample_92, Sample_93, Sample_18, Sample_19, Sample_17, Sample_69, Sample_68, Sample_61, Sample_60, Sample_63, Sample_62, Sample_65, Sample_64, Sample_67, Sample_66, Sample_78, Sample_79, Sample_72, Sample_73, Sample_70, Sample_71, Sample_76, Sample_77, Sample_74, Sample_75, Sample_49, Sample_48, Sample_47
**Reference**	
**Instrument**	S5-0313
**Operation Mode**	Customer mode
**Flow Order**	TACGTACGTCTGAGCATCGATCGATGTACAGC
**Library Key**	TCAG
**TF Key**	ATCG
**Chip Barcode**	DAEI01943
**Chip Check**	Passed
**Chip Type**	530
**Chip Data**	tiled
**Chip Lot Number**	QVB555
**Chip Wafer**	16
**Barcode Set**	IonXpress
**Analysis Name**	Auto_user_S5-0313-18-2019_04_17_AndersonLab_Plate2_169
**Analysis Date**	April 18, 2019, 10:53 p.m.
**Analysis Flows**	0
**runID**	MM39G
**BeadFind Args**	justBeadFind –args-json /opt/ion/config/args_530_beadfind.json
**Analysis Args**	Analysis –args-json /opt/ion/config/args_530_analysis.json
**Pre-BaseCaller**	BaseCaller –barcode-filter-minreads 10 –phasing-residual-filter=2.0 –wells-normalization on
**Calibration Args**	Calibration –num-calibration-regions 1,1
**BaseCaller Args**	BaseCaller –barcode-filter-minreads 10 –phasing-residual-filter=2.0 –num-unfiltered 1000 –barcode-filter-postpone 1 –qual-filter true –qual-filter-slope 0.040 –qual-filter-offset 1.0 –wells-normalization on
**Alignment Args**	tmap mapall -q 50000 ... stage1 map4
**IonStats Args**	ionstats alignment
**Analysis Parameters**	default

Chef Summary

Chef Template Prep Information:

**Chef Last Updated**	April 18, 2019, 8:29 a.m.
**Chef Instrument Name**	242470708
**Chef Operation Mode**	Customer Mode
**Sample Position**	2
**Tip Rack Barcode**	48726009E
**Chip Type 1**	530v1
**Chip Type 2**	530v1
**Chip Expiration 1**	None
**Chip Expiration 2**	None
**Templating Kit Type**	Ion 510
amp; Ion 520	amp; Ion 530 Kit-Chef
**Chef Flexible Workflow**	
**Reagent Expiration**	191130
**Reagent Lot Number**	2012420
**Reagent Part Number**	A34018C
**Reagent Cartridge Serial Number**	None
**Solution Lot Number**	2049981
**Solution Part Number**	A27754C
**Templating Protocol Planned**	(use instrument default)
**Solution Cartridge Serial Number**	None
**Solution Expiration**	191231
**Templating Protocol Executed**	(use instrument default)
**Chef Script Version**	803
**Chef Package Version**	IC.5.10.0
**Start Time**	April 17, 2019, 3:47 p.m.
**Completion Time**	April 18, 2019, 8:29 a.m.

S5 Consumables Summary

**Chip Type**	530v1
**Chip Barcode**	DAEI01943

Product Description	Part Number	Lot Number	Exp. Date	Remaining Uses
Ion S5 Cleaning Solution	100031096	1977166	2019/05/31	2
Ion S5 Sequencing Reagent	INS1012841B	2031538	2019/10/31	1
Ion S5 Wash Solution	100031091B	2033804	2019/10/31	1

iontorrent
by Thermo Fisher Scientific

Software Version

**Torrent_Suite**	5.10.1
**host**	tsvm
**ion-analysis**	5.10.11-1
**ion-dbreports**	5.10.27-1
**ion-gpu**	5.10.0-1
**ion-pipeline**	5.10.10-1
**ion-torrentpy**	5.10.9-1
**ion-torrentr**	5.10.10-1
**S5 Script**	0.1.31
**LiveView**	2389
**DataCollect**	3606
**OIA**	51000
**OS**	35
**Graphics**	97
**Ion_Chef**	IC.5.10.0
